# Multidisciplinary analysis of Italian Alpine wildflower honey reveals criticalities, diversity and value

**DOI:** 10.1038/s41598-021-98876-y

**Published:** 2021-09-29

**Authors:** Valeria Leoni, Luca Giupponi, Radmila Pavlovic, Carla Gianoncelli, Francisco Cecati, Elia Ranzato, Simona Martinotti, Davide Pedrali, Annamaria Giorgi, Sara Panseri

**Affiliations:** 1 Centre of Applied Studies for the Sustainable Management and Protection of Mountain Areas (CRC Ge.S.Di.Mont.), University of Milan, Via Morino 8, 25048 Edolo, BS Italy; 2 Department of Health, Animal Science and Food Safety (VESPA), University of Milan, Via Celoria 10, 20133 Milan, Italy; 3 Fondazione Fojanini Di Studi Superiori, Via Valeriana 32, 23100 Sondrio, Italy; 4 Instituto de Investigaciones en Tecnología Química (INTEQUI), Universidad Nacional de San Luis, Almirante Brown 1455, 5700 San Luis, Argentina; 5 DiSIT-Dipartimento Di Scienze E Innovazione Tecnologica, University of Piemonte Orientale, piazza Sant’Eusebio 5, 13100 Vercelli, Italy; 6 Department of Agricultural and Environmental Sciences - Production, Landscape, Agroenergy (DISAA), University of Milan, Via Celoria 2, 20133 Milan, Italy

**Keywords:** Metabolomics, Biodiversity, Secondary metabolism

## Abstract

Wildflower honeys produced in mountain grasslands are an expression of the biodiversity of these fragile habitats. Despite its importance, the botanical origin of honey is often defined without performing formal analysis. The aim of the study was to characterize six wildflower mountain honeys produced in the Italian Alps with different analytic techniques (SPME–GC–MS, HPLC-Orbitrap, cicatrizing and antioxidant activity) alongside melissopalynological analysis and botanical definition of the production area. Even though the apiaries were in mountain grasslands rich in Alpine herbaceous species, the honey could be defined as rhododendron/raspberry unifloral or raspberry and rhododendron bifloral while the honey produced at the lowest altitude differed due to the presence of linden, heather and chestnut. The non-compliance of the honey could be due to habitat (meadows and pastures) fragmentation, but also to specific compounds involved in the plant–insect relationship, such as kynurenic acid, present in a high quantity in the sample rich in chestnut pollen. 255 volatile compounds were detected as well as some well-known markers of specific botanic essences, in particular chestnut, linden and heather, also responsible for most of the differences in aroma profiling. A high correlation between nicotinaldehyde content and percentage of raspberry pollen (r = 0.853, *p* < 0.05) was found. Phenolic acid and hydroxy-fatty acid were predominant in the chestnut pollen dominant honey, which presented the highest antioxidant activity and the lowest cicatrizing activity, while the flavonoid fraction was accentuated in one sample (rhododendron pollen prevalent), that was also the one with the highest effect on wound closure, although all samples had similar cicatrizing effects apart from the chestnut pollen dominant honey (lowest cicatrizing activity). Our study highlighted the difficulty of producing mountain wildflower honey and the importance of a thorough characterization of this product, also to encourage its production and valorisation.

## Introduction

Honey is defined as a sweet substance produced by *Apis mellifera* L. combining the nectar collected from plants, excretions of plant-sucking insects or sweet excretion of plants with specific substances produced on their own, and subsequent storage in the honeycomb, where the substance ripens, developing specific molecules and lessening water content^1^. Honey is an important food product and the result of interaction between bees as pollinators and the environment, thus providing a key regulating ecosystem service^2^. Honey is mainly classified as monofloral (otherwise unifloral) or multifloral. In the latter case, if the plant species are diverse and very numerous, without any of them assuming a dominant character, they can be described as wildflower honeys.

The botanical origin of honey influences its organoleptic characteristics and finding reliable markers to qualitatively define a certain honey is important for beekeepers. Following the European rules, “honey” may be defined by its floral origin and the consequent organoleptic, physicochemical, and microscopic properties corresponding to that origin^1,3^. According to Community Directive 74/409/EEC (OJEC, 1974)^3^ it is not compulsory to specify a botanical origin, but the European Commission has adopted a proposal to amend this Directive to harmonise the European Union market. Therefore, classification of honey is very often performed commercially without carrying out melissopalynological analysis. This analytical technique is the most recognized method to define the floral origin of honey based on the identification and quantification of pollen percentages by microscopic examination^4^. In such cases, the botanical origin of honey is based on the claims of local beekeepers, and the determination of honey origin is performed by sensory analysis (often subjective) or by considering the predominant flowers surrounding the hive.

In the case of wildflower honeys produced in the mountain regions of the Italian Alps, this can lead to ambiguity in defining the qualitative characteristic of these products, since mountain areas are fragile territories subject to climatic and anthropic changes. As regards climate change, the altitudinal range of the habitat of many alpine flower species is increasing and many plants have a more limited areal, with the alpine biome in general decreasing^5^. As regards anthropic changes, in the last decades, and even today, the European Alps have been subject to abandonment by the population^6^. This anthropic process has caused agricultural practices to be progressively neglected^7^, and forest expansion in areas once occupied by mountain grasslands (meadow and pasture)^8,9^ reducing the habitat for wild and domesticated bees. Honey produced from mountain grassland is then defined “presidium” following the rules of the Slow Food Foundation for Biodiversity^10^.

Based on melissopalynological analysis, the term ‘‘unifloral honey’’ defines honey produced mainly from one vegetal nectar source, represented by more than 45% of the total pollen content^11^. However, pollen analysis can be not enough descriptive of some honey types obtained from plant with underrepresented or overrepresented pollen, such as *Castanea sativa* Mill.^12^, and in any case melissopalynology requires very expert analysts^13^.

A detailed chemical characterization of honeys, although more rarely performed, can provide an additional tool to describe them. Some studies have suggested possible correlations between floral origin and flavonoid profiles^14^ and other than flavonoids, honey has highly characteristic aromas, indicating the presence of volatile substances derived from nectar^15,16^. Volatile substances are the main factors responsible for aroma, which together with other factors, such as taste, and physical factors, contribute to the flavour^17^. Only plant derived compounds and their metabolites (terpenes, norisoprenoids and benzene compounds and their derivatives) must be employed to discriminate among floral origins of honey^18^. As well as being an important food product, honey has been used as natural sweetener and natural remedy for centuries^19^ and its chemical composition, depending on the floral source, can attribute higher or lower antioxidant, cicatrizing or antibacterial activity to the various honeys^20–22^.

The aim of this work is to characterize six honeys defined commercially as wildflower mountain honeys produced in a Valley of the Italian Alps (Camonica Valley) using different analytic techniques. From the starting point of the floristic analysis of the area considered, honey samples were then analysed through chromatographic techniques in addition to melissopalynological analysis. Moreover, some bioactivities of honey were tested to get a complete overview regarding these kinds of honey and to identify specific characteristics for their definition and valorisation.

## Materials and methods

### Sampling areas and vegetation analysis

**Figure 1:**
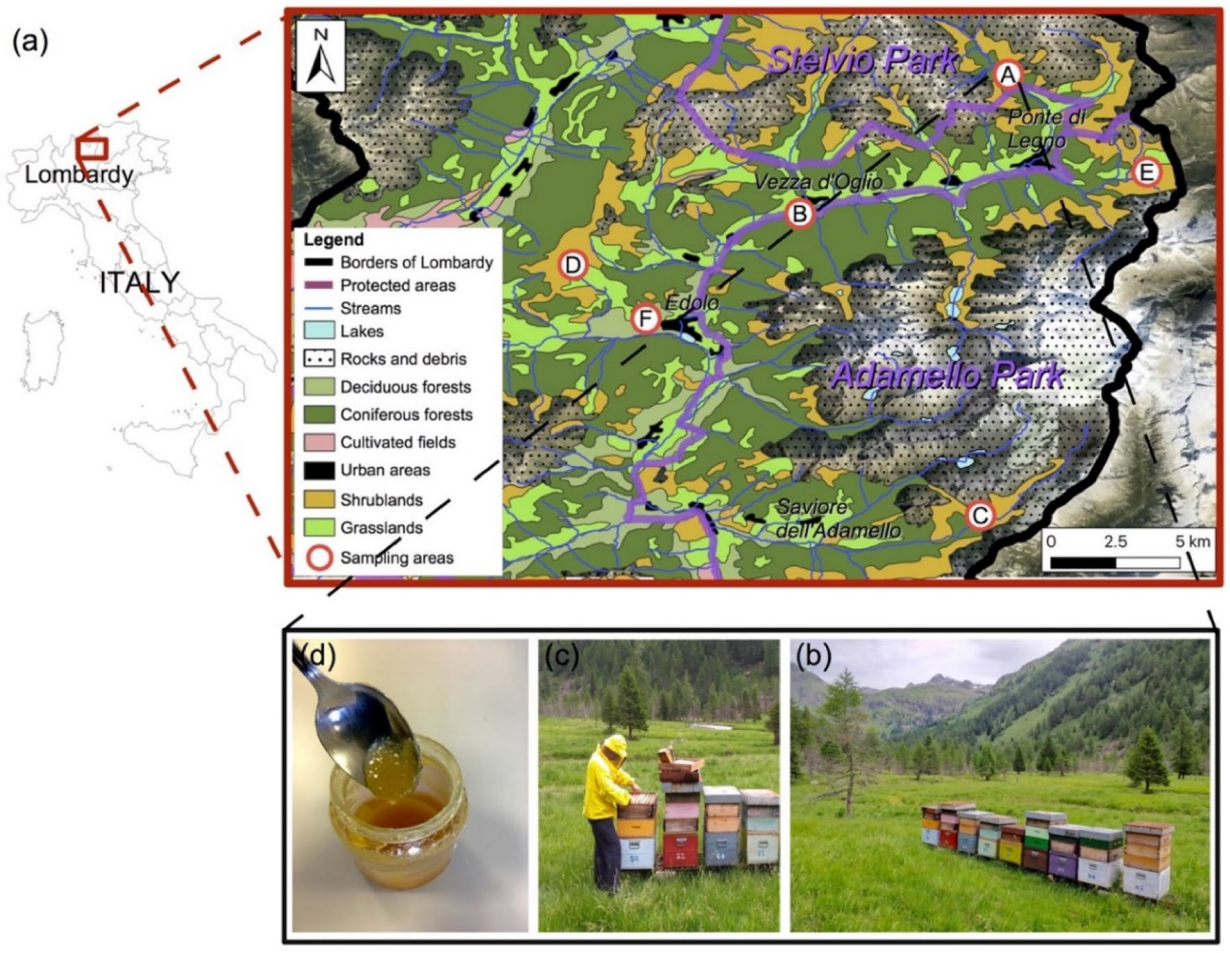
Map of the upper Camonica Valley with the six areas (A, B, C, D, E and F) where honey was produced and sampled (**a**) and location of the apiaries (**b**), collection of honey (**c**) and honey (**d**) produced in area A. The map was generated using ArcGIS Desktop 10.5 (Esri, Redlands, CA, USA, https://www.esriitalia.it/prodotti/la-nostra-offerta/piattaforma-esri/arcgis-10-5).

Six bee stations in the mountain areas of high Val Camonica (Southern Alps, Northern Italy, Lombardy region) were chosen for sampling in the spring of 2020 (Fig. 1). Three production areas are inside the Adamello Regional Park and one is in the Stelvio National Park. Phytosociological relevés were performed to collect floristic data about the study area in July, during the blooming season. Later, during the second week of August, beekeepers performed honey extraction to produce the wildflower variety. Finally, honey was sampled from the decanters during the first week of September with the appropriate permission for the collection of samples.

The hives (8–15 for each production area) were positioned in areas with different elevation, slope, and exposure (Table 1) in order to produce wildflower honey. Production area F is located below 1000 m a.s.l. while the others are located at higher elevations.

**Table 1:** - Location of the six production/sampling areas.

Sampling area	Municipality	Latitude N	Longitude E	Elevation (m a.s.l.)	Slope (°)	Exposure (°)
A	Ponte di Legno—Messi Valley	46° 18′ 38″	10° 29′ 15″	1683	6	70
B	Vezza d'Oglio	46° 14′ 29″	10° 25′ 21″	1075	8	167
C	Saviore dell'Adamello	46° 05′ 03″	10° 29′ 10″	1626	14	185
D	Edolo—Alpe Mola	46° 11′ 57″	10° 17′ 54″	1750	9	148
E	Ponte di Legno—Tonale Pass	46° 15′ 13″	10° 33′ 40″	1805	16	352
F	Edolo—Loc. Plerio	46° 09′ 05″	10° 20′ 09″	850	36	225

According to the classification of the ecoregions of Italy^23^, high Camonica Valley is within the Central and Eastern Alps section (Alpine Province, Temperate Division) and has a sub-continental climate (average annual rainfall: 900–1100 mm; average annual temperature: 4–10 °C)^24^.

Vegetation types of Camonica Valley are many and diversified mainly on the basis of elevation range. In the submontane areas of high Camonica Valley (at elevations below 1000–1200 m a.sl.) there are broad-leaved woods of *Cytiso nigricantis-Querco petraeae sigmetum* characterized by trees such as durmast oak (*Quercus petraea* (Matt.) Liebl.), chestnut (*Castanea sativa* Mill.), hazel (*Corylus avellana* L.) and ash (*Fraxinus excelsior* L.), and shrubs such as *Calluna vulgaris*, *Genista germanica* and *Chamaecytisus hirsutus*^25^. There are also meadows of *Arrhenatherion elatioris* phytosociological alliance that are grasslands regularly mown twice a year and rich in species such as *Arrhenatherum elatius* (L.) P. Beauv. Ex J. Presl & C. Presl, *Pimpinella major* (L.) Huds., *Lolium perenne* L., and *Schedonorus pratensis* (Huds.) P. Beauv., *Phleum pratense* L.^26^.

In areas between 1200 and 1700 m a.s.l. there are mostly coniferous forests of *Calamagrostio arundinaceae-Piceo excelsae sigmetum*^25^ dominated by spruce (*Picea abies* (L.) H. Karst.) and larch (*Larix decidua* Mill.) with the presence of shrubs (such as *Rubus idaeus* L., *Vaccinium myrtillus* L., *Calluna vulgaris* and *Alnus viridis* (Chaix) DC.) and herbs (such as *Oxalis acetosella* L., *Solidago virgaurea* L. and *Luzula nivea* (L.) Lam. et DC.). In addition, there are mountain meadows of *Trisetetum flavescentis* association and acidophilus pastures of *Siversio-Nardetum*^8,25^.

At higher altitudes, especially beyond the treeline (2050–2200 m a.s.l.), there are mainly alpine acidophilus grasslands of *Caricetea curvulae* class^26^ with small shrubs such as *Thymus* spp., *Rhododendron ferrugineum* L., *Rubus idaeus*, *Vaccinium myrtillus*, *V. vitis-idaea* L., *Calluna vulgaris* and *Juniperus communis* L.^25^. These shrubs (and some trees such as larix and spruce) are colonizing the montane and sub-alpine grasslands abandoned by man^8,9^.

Finally, near urban areas and rural areas there are ornamental/exotic or cultivated plants (*Malus* spp., *Pyrus* spp., *Ficus carica*, *Prunus avium*, *Robinia pseudoacacia*, *Rosa* spp.) as well as hygrophilous species such as willows (*Salix* spp.) and alder (*Alnus* spp.) along streams.

Floristic data of the vegetation types close to the hives were collected performing phytosociological relevés in accordance with the method of Braun-Blanquet^27^. In each honey production area (Fig. 1) 4 relevés were carried out in June–July 2019 on a surface of 100 m^2^ (10 × 10 m plot), starting from 5 m away from the apiaries and proceeding towards the four cardinal points. Phanerogam species were identified using “Flora d’Italia” dichotomous keys of Pignatti^28^ and their coverage was estimated using the abundance/dominance scale of Braun-Blanquet^27^: r, rare species in the relevé; + , coverage < 1%; 1, coverage 1–5%; 2, coverage > 5–25%; 3, coverage > 25–50%; 4, coverage > 50–75%; 5, coverage > 75–100%. The data of the relevés were arranged in a matrix (relevés x species) where abundance/dominance indexes of the species were converted into the percentage of coverage in accordance with^9^ to perform statistical analysis.

Multidimensional scaling analysis (MDS) was performed on the matrix of the relevés to highlight floristic differences among the relevés and the honey production areas, and to identify the main ecological gradients that determine these differences. MDS was carried out considering the chord distance^29^ and was performed using the “vegan” package of R 3.5.2. software^30^. The scientific names of the species mentioned are in accordance with^28^.

### Melissopalynological analysis

Melissopalynological analysis was performed according to the techniques proposed by the International Commission for Bee Botany (ICBB) for qualitative melissopalynological analysis^4^ opportunely modified as described below. Organoleptic characteristics were also concisely analysed.

Slide preparation was performed according to the following method: 12 g of honey were weighted in a 50 mL test tube with conical bottom and dissolved with 40 mL of distilled water. The solution was centrifuged for 15 min at 3000 rpm, and then the liquid was separated from the sediment by pouring or aspiration. To eliminate the sugars present, the sediments were dissolved again in 10 mL of distilled water and centrifuged again for 5 min at 3000 rpm and the supernatant was eliminated by pouring gently. The sediment was carefully dispersed with a Pasteur pipette, transferred to a glass slide, and distributed as evenly as possible over an area of ​​about 1 cm^2^. The glass slide was left to dry at 40 °C. When the sediment was dry, it was included in a drop of glycerol gelatine, previously dissolved in a water bath, and covered with a coverslip.

The microscopic analysis of honey sediment composition provides the percentage of specific pollen observed by microscopic comparison with known pollen grains. Pollen grain counting was performed according to the method described in^31^. If the sediment contained a high percentage of overrepresented pollen, a second count excluding the over-represented pollen was performed in order to determine the relative abundance of other pollen types more precisely. The pollen types present in the honey samples were identified, counted, and classified, according to their percentages, as dominant pollen (more than 45% of the total pollen grains counted), secondary pollen (from 16 to 45%), important minor pollen (from 3 to 15%), minor pollen (less than 3%) or sporadic pollen (less than 1%)^32^.

MDS was performed on the matrix of the melissopalynological data to highlight the differences among the honey samples using R.

### Bioactivity

As regards bioactivity, antioxidant (Free Radical Scavenging Activity, FRSA) and cicatrizing properties were examined through DPPH and HaCaT cell culture tests.

The DPPH radical scavenging activity of honey samples was determined according to the method of Brand-Williams^33^ modified as in^34^. The DPPH solution in methanol (0.06 mM) was prepared daily, and 2.7 ml of this solution was mixed with 0.3 ml of 50% (w/v) honey solution. The mixture was shaken vigorously and left to stand for 60 min in the dark (until stable absorption values were obtained). The reduction of the DPPH radical was determined by measuring the absorption at 517 nm. The experiment was carried out in triplicate. Radical scavenging activity (RSA) was calculated as a percentage of DPPH discoloration using the equation:

$${\text{Radical}}\,{\text{Scavenging}}\,{\text{Activity}}\,\left( \% \right) = \left[ {\left( {{\text{A}}0{-}{\text{A1}}} \right)/{\text{A}}0} \right]*{1}00$$

where A1 is the absorbance of the sample solution and A0 is the absorbance of the radical blank using distilled water. The honey samples were confronted with a manuka honey sample.

Cicatrizing activity was performed on a HaCaT cell culture. HaCaT cells are immortalized human skin keratinocytes that mimic many properties of normal epidermal keratinocytes. These cells are not invasive and can differentiate under experimental conditions^35^. Cells were cultured in a Dulbecco’s modified Eagles’ medium (DMEM) supplemented with 10% fetal bovine serum (FBS; Euroclone, Pero, Italy), 1% glutamine, 100 U/ml penicillin and 100 mg/ml streptomycin.

Scratch wounds were made in confluent layers HaCaT cells by using a sterile 2–20 µL pipette tip^36^. After washing away detached cells, cultures were rinsed with complete medium in the presence of honeys (0.5% w/v) for 24 h. After exposures, cells were fixed in 3.7% formaldehyde in PBS for 30 min and stained for 30 min at room temperature with 0.1% toluidine blue.

The space between the wound edges was measured at T0 (at wounding) and at T24 (end of exposure) by using an inverted microscope (Leica Microsystems) equipped with a digital camera.

NIH ImageJ software was used to analyse the digitalized pictures. Wound closure rate was calculated as the difference between wound width at T0 and at T24. The value of the control was set at 100 percent to obtain wound closure rate %.

### Phytochemical analysis

The phenolic and flavonoid profile was obtained through HPLC-Q-Exactive-Orbitrap-MS metabolomic analysis of the six samples. The phenolic compounds were extracted by the following SPE procedure: 2 g of honey was diluted in 5 mL of 0.01 M HCl and 12 μL of IS (4-Fluorobenzoic acid) was added. The SPE cartridges (STRATA X 33) were preconditioned with 3 mL of methanol, followed by 5 mL of water. The honey samples were then introduced into the cartridges at a drop wise flow rate. After loading, the SPE cartridge was left under vacuum for 3 min to completely dry out. The phenolic compounds retained by the cartridges were finally eluted with 3 mL of elution solvent (methanol: acetonitrile = 2:1). The eluent was filtered by 0,45 μm syringe filter and subsequently diluted with 0.1% of formic acid (1:1). 10 μL was introduced into HPLC–Q-Exactive-Orbitrap–HRMS instrumentation. Untargeted metabolomic approach was performed according to the recently developed strategy for propolis^37^. All analyses were performed in triplicate.

Heatmap and Principal Components Analysis (PCA) were performed on the metabolomic data in order to highlight the differences among honey samples.

The volatile profile was obtained through Headspace Solid-Phase Microextraction (HS SPME) coupled to Gas Chromatography–Mass Spectrometry (GC–MS) analysis of Volatile Organic Compounds (VOCs).

All the samples were prepared by weighing exactly 5 g of honey in a 20 mL glass vial along with 100 μL of the IS (4-Methyl-2-pentanone, 2 mg/L in 2-propanol). Each vial was fitted with a cap equipped with a silicon/PTFE septum (Supelco, Bellefonte, PA, USA) and passed in an ultrasonic bath for 10 s at 30 °C., fitted with cap and equipped with silicon/PTFE septa (Supelco, Bellefonte, PA, USA). At the end of the sample equilibration period (1 h), a conditioned (1.5 h at 280 °C) 50/30 μm Divinylbenzene/Carboxen/polydimethylsiloxane (CAR/PDMS/ DVB) StableFlex fibre (Supelco, Bellefonte, PA) was exposed to the headspace of the sample for extraction (120 min) by CombiPAL system injector autosampler (CTC analytics, Switzerland). The fibre and the time of extraction used in this study were selected after preliminary study. The best adsorption of analytes was obtained using CAR/PDMS/DVB and 120 min as extraction time. The extraction temperature of 25 °C was selected to prevent possible matrix alterations (oxidation of some compounds, particularly aldehydes and furans). The vials were maintained on a heater plate (CTC Analytics, Zwingen, Switzerland) to keep temperature constant during analysis.

GC–MS analysis was performed using a Trace GC Ultra (Thermo-Fisher Scientific, Waltham, MA, USA) Gas Chromatograph coupled to a Trace DSQII quadrupole mass spectrometer (MS) (Thermo-Fisher Scientific, Waltham, MA, USA) and equipped with an Rtx-Wax column (30 m; 0.25 mm i.d.; 0.25 μm film thickness, Restek, USA). The oven temperature program was from 35 °C, hold 8 min, to 60 °C at 4 °C/min, then from 60 to 160 °C at 6 °C/min, and finally from 160 to 200 °C at 20 °C/min, hold 20 min. Carryover and peaks originating from the fibre were regularly assessed by running blank samples. After each analysis, fibres were immediately thermally desorbed in the GC injector for 5 min at 250 °C to prevent contamination. The injections were performed in splitless mode (8 min). The carrier gas was helium at a constant flow of 1 mL/min. The transfer line to the mass spectrometer was maintained at 230 °C, and the ion source temperature was set at 250 °C. Mass spectra were obtained by using a mass selective detector with electronic impact at 70 eV, a multiplier voltage of 1456 V, and by collecting the data at a rate of 1 scan s^−1^ over the m/z range of 35–350. An alkanes mixture (C8–C22, Sigma R 8769, Saint Louis, MO, USA) was run under the same chromatographic conditions as the samples to calculate the Kovats Retention Indices (RI) of the detected compounds. Compounds were identified by comparing the Kovats retention indices with literature data and through the National Institute of Standards and Technology (NIST) MS spectral database as in previous research^38–40^. Volatile compound measurements from each headspace of honey extracts were carried out by peak area normalization (expressed in ppb). All analyses were performed in triplicate.

Heatmap was performed on the VOCs data to highlight the differences among honey samples.

### Ethics declaration

There are no special ethical concerns arising from the use of animals or human subjects.

## Results and discussion

**Figure 2:**
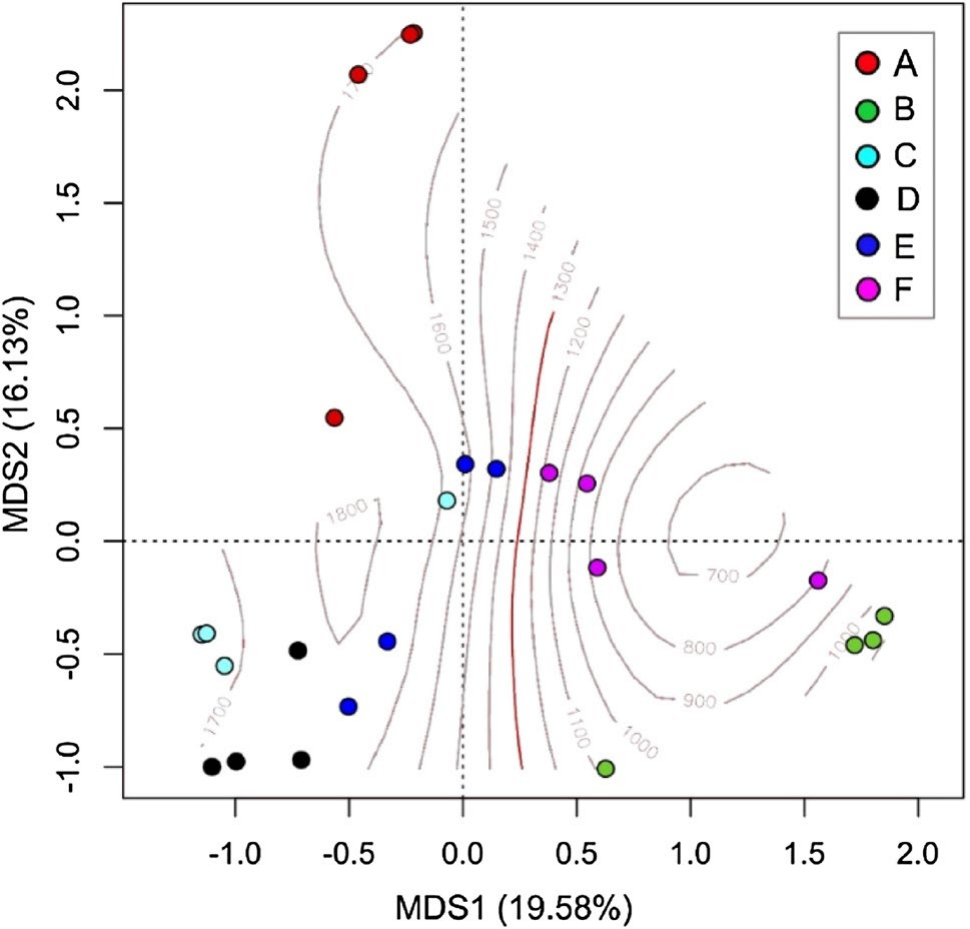
MDS of the phytosociological relevés. Capital letters indicate the six sampling areas, the 1.300 m a.s.l. contour line that separates sub-montane belt and high mountain belt vegetation is highlighted in red.

From the phytosociological relevés performed in each sampling area it is evident that hives were positioned in grasslands rich in Alpine herbaceous species (Table S1). In fact, among the 169 identified species, 85% were herbaceous species common in meadows (of *Arrhenatherion elatioris* and *Triseto flavescentis-Polygonion bistortae* phytosociological alliance) and acidophilus pastures (*Siversio-Nardetum*). 15% of the species were trees and shrubs (not abundant in the floristic relevés of the apiary areas considered), including some of beekeeping interest such as: *Rhododendron ferrugineum*, *Castanea sativa* and *Rubus idaeus*. From the MDS biplot (Fig. 2) elevation is the main ecological variable that differentiates sampling areas. In particular, the relevés of stations B and F are characterized by a floristic composition which is different from the areas at higher elevation (characterized by a higher presence of microthermal alpine species). This is due to the separation between the sub-montane belt and the high mountain belt vegetation on the 1.300 m a.s.l. line in the study area^25^.

**Figure 3:**
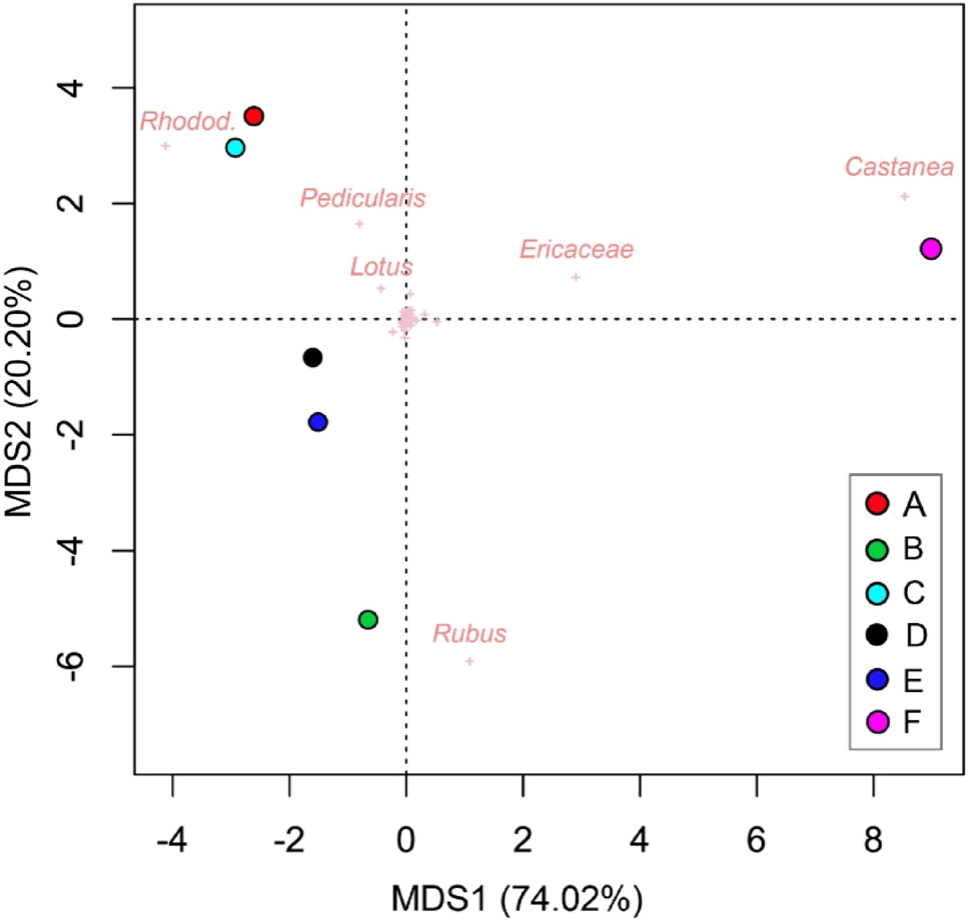
MDS of the melissopalynological analysis of the six samples (dots) of mountain wildflower honeys produced in the stations considered. The crosses are the pollens found in the honey samples, the most important are indicated.

Although the beehives were positioned in mountain grasslands, melissopalynological analysis presented a different picture. The pollen of numerous species detected through the floristic relevés were found in the honey samples via melissopalynological analysis, although the latter did not totally overlap with the floristic characterization of the area, in particular from a “quantitative” point of view. In fact, the floristic relevés showed a relative richness of herbaceous species (Table S1) peculiar of mountain grasslands that would seem promising for the production of wildflower honeys. Conversely, in the melissopalynological analysis the species considered interesting but not predominant in the botanical description were relevant (Fig. 3 and Table S2).

The premises to produce wildflower honey is that the botanical species contributing must be different and sometimes very numerous, without any of them assuming a dominant character. However, this was not fully evident in our research: although it was possible to identify more than seventy species through melissopalynological analysis and even more through the floristic characterization of the areas, most of them were defined as minor or sporadic pollen (Table S2). Even though apiaries were in mountain grasslands, the most relevant role was played by some woody species/shrubs: *Rubus* (presumably *Rubus idaeous* L., identified in the floristic relevés) and rhododendron (*Rhododendron ferrugineum* L.) for the mountain/subalpine belt and *Castanea* and *Ericaceae* (heather) in the submountain belt. Following the rules to define ‘‘unifloral honey’’, three of the wildflower honeys could be defined unifloral or bifloral:

- Rhododendron unifloral: honey A (Rhododendron 47.18%), and honey C (Rhododendron 62.93%);
- Raspberry unifloral: honey B (Rubus 67.12%)
- Raspberry and Rhododendron bifloral: honey D (Rhododendron 34.27% and Rubus 34.74%) as well as honey E (*Rubus* 44.25%, Rhododendron 34.14%).

Honey F, due to the contribution of pollen from *Tilia* genus (that was detected only in this sample as an important sporadic pollen, 3.5%) *Castanea* (96.4% in honey F, but it should be noted that chestnut pollen is an overrepresented pollen) and in the second count *Ericaceae* (32.45%, that was considered a secondary pollen together with *Rubus*, with a percentage of 38.59% in honey F) differed from the other honeys (Fig. 3).

*Rubus* pollen was anyway present in good amounts in all the samples considered, and was a dominant pollen in honey B, a secondary pollen in honeys C, D, E and F and a minor pollen in honey A. *Sorbus* and *Tilia* pollens were detected only in honey F, while no rhododendron was detected in honey F. Honey D was characterized by a percentage higher than the “rare pollen” category of some important alpine essences, such as *Liliaceae*, *Centaurea*, *Campanulaceae*, *Anthyllis* f., *Polygonum bistorta*, *Lotus alpinus* and *Potentilla/fragaria* (Table S2).

Although wildflower honeys are intrinsically characterized by a high variability compared with unifloral honey, this shows the importance of the formal characterization of honey to obtain a product which satisfies consumer expectations, and it was demonstrated that the botanical origin of honey cannot be based on the claims of local beekeepers by considering the predominant flowers surrounding the hive.

Although honeybees are considered supergeneralists in their foraging choices, there are certain key species or plant groups that are particularly important in honeybee foraging^2^, and many were identified in the botanical characterization of the area, including *Rubus idaeus* L., *Calluna vulgaris* L., rhododendron and some present in the broad-leaved woods mentioned such as chestnut (*Castanea sativa* Mill.) or plants of *Tilia* genus. In the research work by Hawkins et al.^2^, *Rubus fruticosus* L. was among the frequently found species and tree pollen belonging to *Castanea sativa* L. as well as, for example, species of *Malus*, *Salix* and *Quercus* spp, was frequently seen. These kinds of preferences could relate to the ease of availability and abundance of the plant, the quality and abundance of the nectar and pollen and/or specific nutrients or trace elements provided by these species or neurological aspects (as will be discussed further). As referred by beekeepers, over the last decades the production of mountain wildflower honey, that often does not meet the characteristics expected and presents flavours that are reminiscent of other kinds of honey such as rhododendron or linden or chestnut, is becoming more and more critical and this was absolutely confirmed by this study.

This could be linked to the fragmentation of an important habitat of the Alps—mountain grasslands (meaning pastures and meadows) for anthropic and climatic reasons^8,9^. Honeybees from the same colony forage across areas spanning up to several hundred square kilometres, and at linear distances as far as 9 km from the hive^41^. Onlooker bees are those in charge of finding nectar sources and of giving instructions to the employed bees, the other foraging bees, that communicate the necessity to look for new resources of food to the onlookers through continuous dance communication^42^. Among the onlookers, there is a difference between the bees that scout for different nectar sources or recruit to well known floral resources^43^ and there is an optimal ratio of scouts to recruits, for the most effective collective foraging^41^. However, this balance may change based on the structure of the landscape in which the bees forage for food^44–46^. Theoretical models^47,48^ and empirical tests^49^ suggest that when resources are concentrated into a small number of highly rewarding patches, colonies perform best with few scouts and many recruits, while when resource patches are small, evenly distributed, and easy to locate, successful colonies invest more in scouting than in recruitment. This is strictly linked to climate and social changes in the mountains: mountain grasslands are no longer evenly distributed and easily localizable, as they are scattered among expanding areas of shrublands and forests^9^ and, for the above-mentioned reasons, it is more efficient for the colony to invest in more recruiters than scouters, as recruiters will identify a small number of highly rewarding patches, such as raspberry or rhododendron shrublands or linden and chestnut woods, that are highly rewarding and very different in quality.

**Figure 4:**
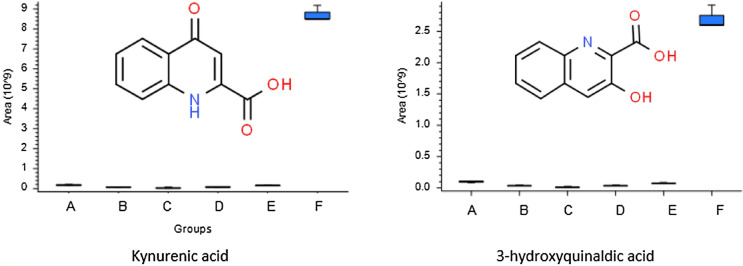
Kinurenic acid and 3-hydroxyquinaldic acid structure and content in the six honey samples, performed in triplicate. The box diagram representing the median with distribution interval between 25 and 75%.

This overlaps with individual and collective honeybee behaviour driven by proximate physiological mechanisms that involve the tryptophan metabolism via kynurenine pathway that is one of main neuroprotective mechanisms. In this research, many of the differences/similarities among the samples might be attributed to metabolic alterations within this pathway, represented by relative amounts of kynurenic acid. However, different quinoline structures have also been identified (Fig. 4). Neurotransmitters play a central role in several of the biological processes that honeybees require to perform activities such as foraging behaviour^50^. A considerable amount of literature highlights the involvement of the neuroprotective kynurenine pathway (KP) final product kynurenic acid (KinA) in the regulation of the stress-related hormone dopamine in the honeybee as well as in other animal species^51,52^. The major known source of dietary KynA are pollen and nectar produced by sweet chestnuts^53,54^ and it has been verified that this compound is found in high concentrations in chestnut flowers^55^. This is coherent with the results of this study: chestnut pollen was found in honey F, produced in the lower station where chestnuts also appear in the floristic relevés, and KynA was found to be a dominant compound in honey F. Interestingly, chestnut pollen was found as sporadic pollen in all the other samples, even those produced in the highest apiary stations (Table S2).

**Figure 5:**
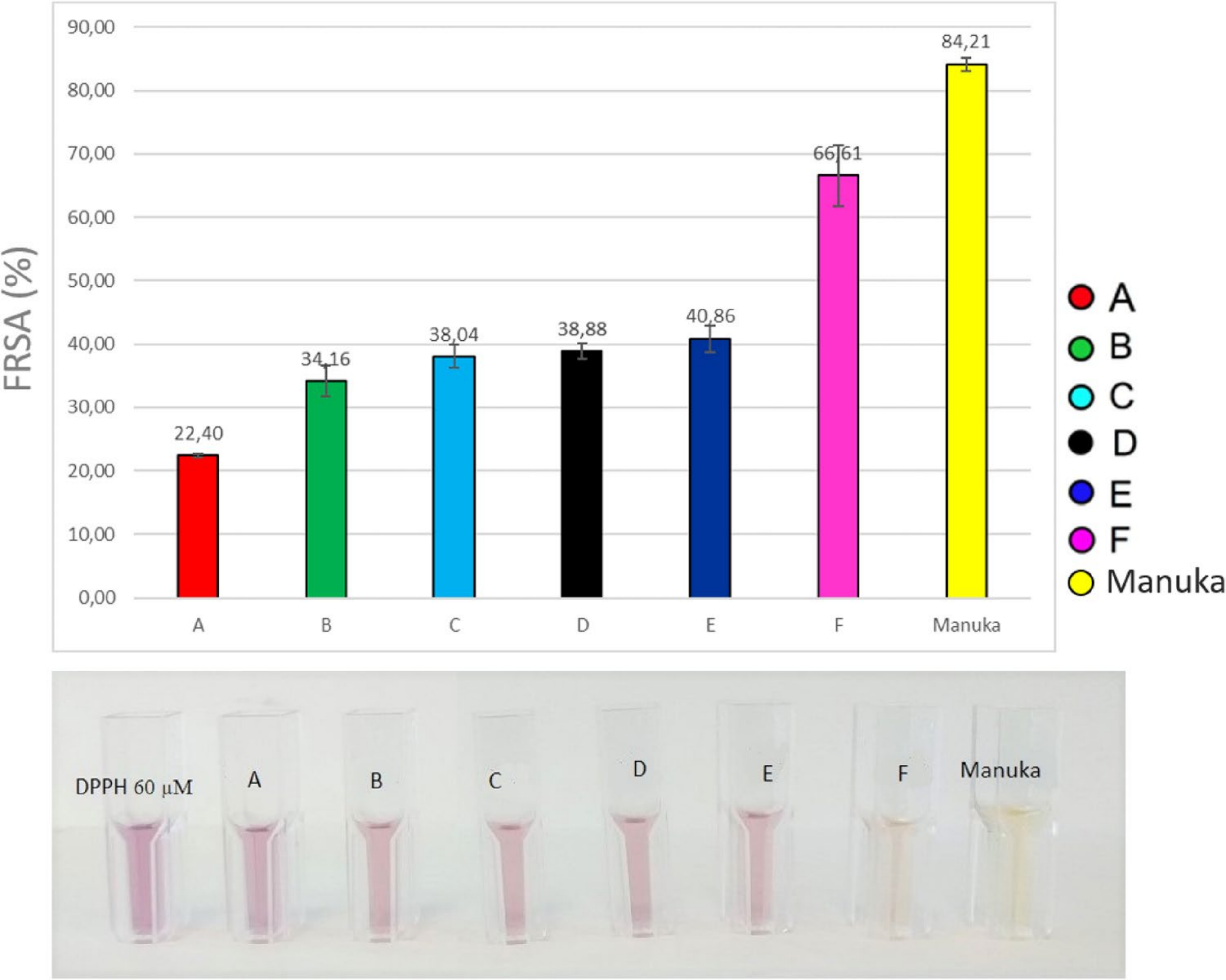
Results of the FRSA test. Capital letters represent the six honey samples considered. Manuka honey was used as a control*.*

Further, KinA may possess positive properties in a number of pathologies of the gastrointestinal tract, especially colitis, colon obstruction or ulceration^56,57^. It has been proposed that KinA may also possess antioxidative properties^56–59^. This was confirmed by this study, since the wildflower honey with a high component of chestnut pollen was the one with the highest antioxidant properties at the FRSA test (66.61 ± 4.77%), even if lower than manuka honey (84.21 ± 1.04%), a dark honey that is a well-known nutraceutical product and has recently attracted attention for its biological properties, especially for its antioxidant and anti-microbial capacities^60^. Honey A showed the lowest power (22.40 ± 0.28%) while the other honeys ranked around 40% (Fig. 5). Interestingly, metabolomic analysis revealed the presence of 3-hydroxyquinaldic acid (Fig. 4), which is a kynurenic acid isomer and, although its function has not been elucidated in detail, a few literature data indicate its role as a precursor of naturally occurring peptide antibiotics from the quinomycin family^61^.

**Figure 6:**
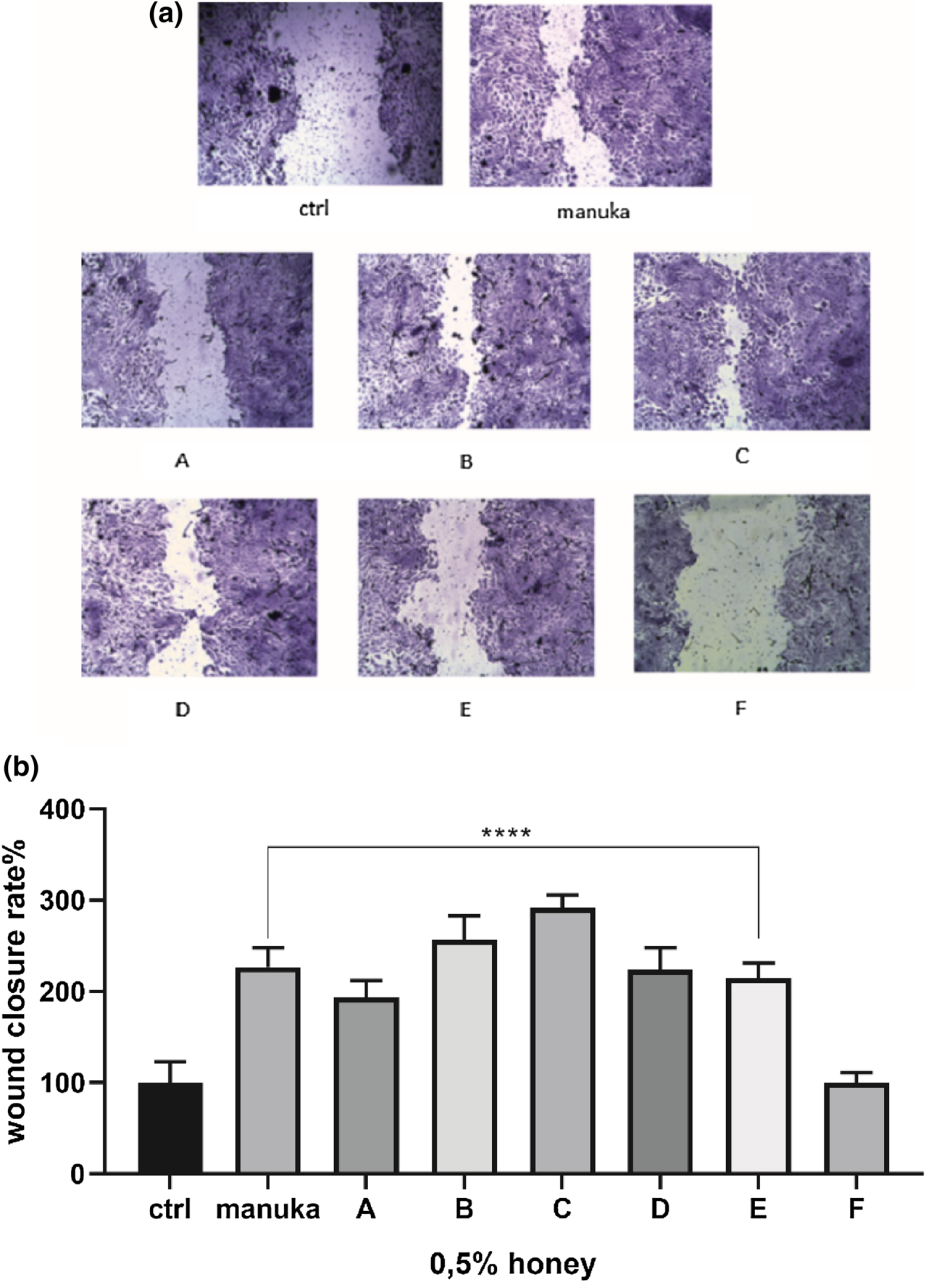
The scratch wound test in keratinocytes, HaCaT cells, exposed to honeys. (**a**) The digitalized pictures of scratched cells after 24 h exposure to 0.5% (w/v) of honeys. (**b**) The closing percentage wound values after 24 h exposure. Statistics on bars indicate differences compared to the control (CTRL) condition determined by a One-Way ANOVA followed by Dunnett’s test (****p < 0.0001).

In order to evaluate the ability of honey to induce wound closure, a scratch wound assay was performed (Fig. 6)^62^. Scratch assay creates a gap in confluent keratinocyte monolayer to mimic a wound. It has already been demonstrated that honeys are able to induce wound closure^63^ to different extents depending on honey origins and properties.

The six honeys were compared to manuka honey^60^. Results suggested that honeys A-E induce a significant effect on wound closure, allowing a positive action on the wound edge closure after 24 h of exposure. The results were comparable to that obtained with manuka at the same concentration. Only sample F did not induce a relevant effect if compared to control condition (i.e. complete medium).

The efficacy of natural honey in wound care has been attributed to its anti-inflammatory activity, for which specific flavonoids and polyphenols, as for example kempherol^64^, are considered to be partly responsible. Honey C was the richest in terms of some specific flavonoids, among which tricin, luteolin, pectolinarigenin, naringenin and kempherol (Figure S1), together with a specific glycoside (dihydroxyfenchone 6-O-d-glucoside). Majtan et al.^65^ discovered two other flavonoids in aqueous extract of honey, one of which, kempherol, suppresses the activity of TNF-α-induced Multiple Medical Problems (MMP)-9 expression in HaCaT and according to Budovsky et al. in^66^ in terms of plant phytochemicals, the activity of alkaloids, flavonoids, terpenes, and glycosides in promoting wound healing, has been better researched than other bioactive plant compounds. It is known that the chemical composition of honey predominantly depends on its botanical source^67^. Among all substances in honey, the ones that are the most depending on floral sources are phenolic compounds, that are also important for their antioxidant activity^68^ while processing, handling, and storage have a lesser impact on the phenolic profile and composition of honey. Phenolic acids and flavonoids can be then considered important markers for the identification of botanical origin for different honey^69,70^.

**Figure 7:**
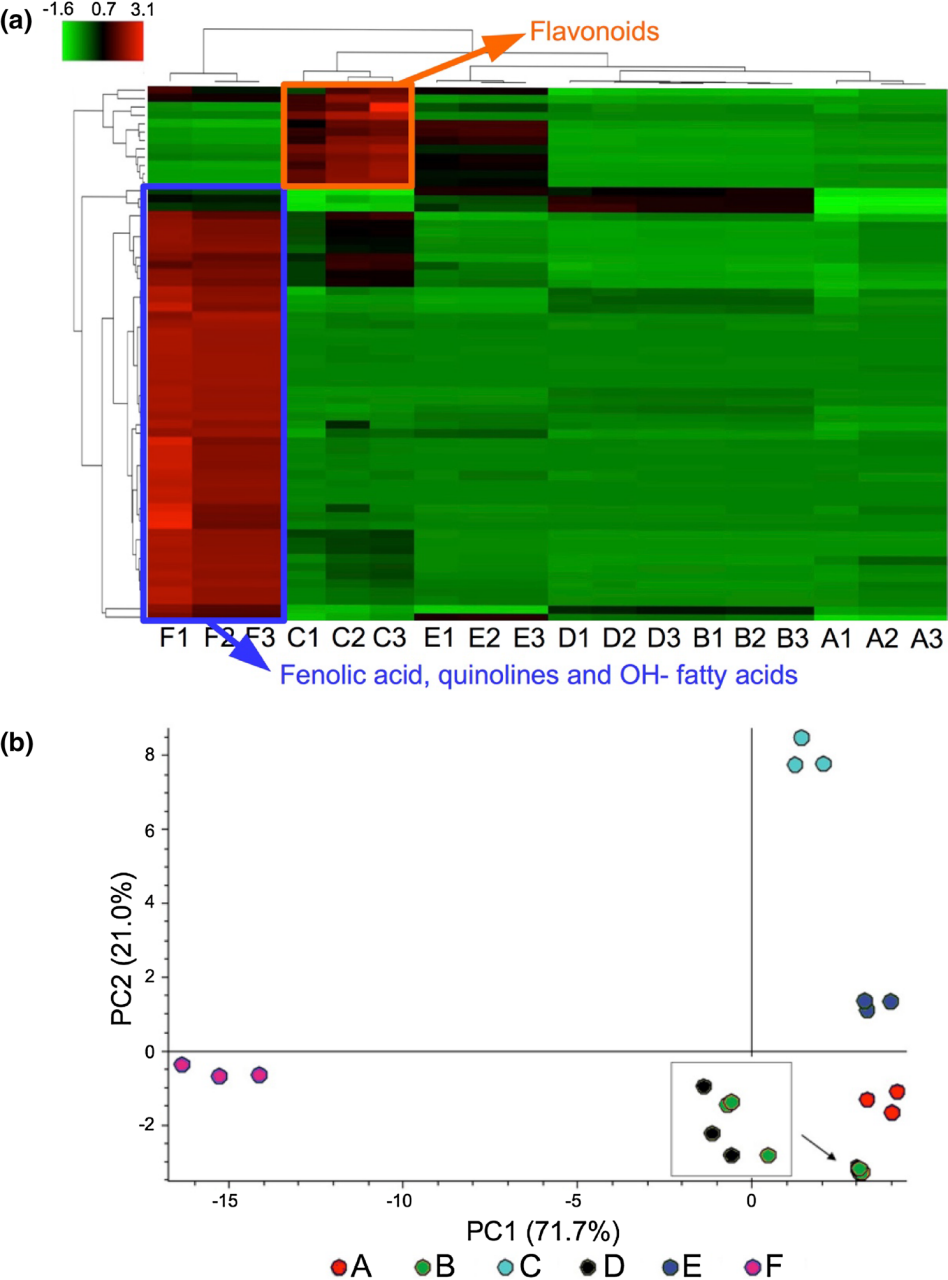
Hierarchical cluster analysis (**a**) and biplot of PCA (**b**) reflecting the differences between compounds revealed in honey samples (red region in heat map for F present prevalently phenolic acid and hydroxy fatty acids profile while the red region for C stands for reach flavonoids fraction). Capital letters indicate the samples and the number the analytical repetitions. Heat-map (cells, exposed to honeys) was generated using R 3.5.2. software^30^.

Heatmaps, PCAs and pathway molecular networks, very different but complementary visualization techniques, confirmed and complemented one another providing an additional perspective to recognize the results from honey phenolic compounds analysis (the total ion current chromatograms obtained for each honey samples are showed in Figure S3). The heatmap and PCA (Fig. 7) were performed to compare the overall distribution and variation within and between the honey samples. The PCA and hierarchical cluster analysis indicated that the different floral origins caused significant metabolic changes in the honey samples. A clear separation was observed between sample F (chestnut honey) and other samples. Also, C (prevalently rhododendron) and E (rubus and rhododendron) exhibited specific profiles regarding flavonoid composition. On the other hand, samples B and D, despite the different *rhododendron/rubus* ratio, exhibited a substantial similarity in their phenolic compound profiles.

The most important compounds found are presented in Table S4 and the possible metabolic transformation is presented in Figure S2. The flavonoid fraction was accentuated in C and it corresponds to the heatmap results while phenolic acid and hydroxy-fatty acids were predominant in F. For each type of honey, the most important candidates for differentiation are presented in Figure S1.

**Figure 8:**
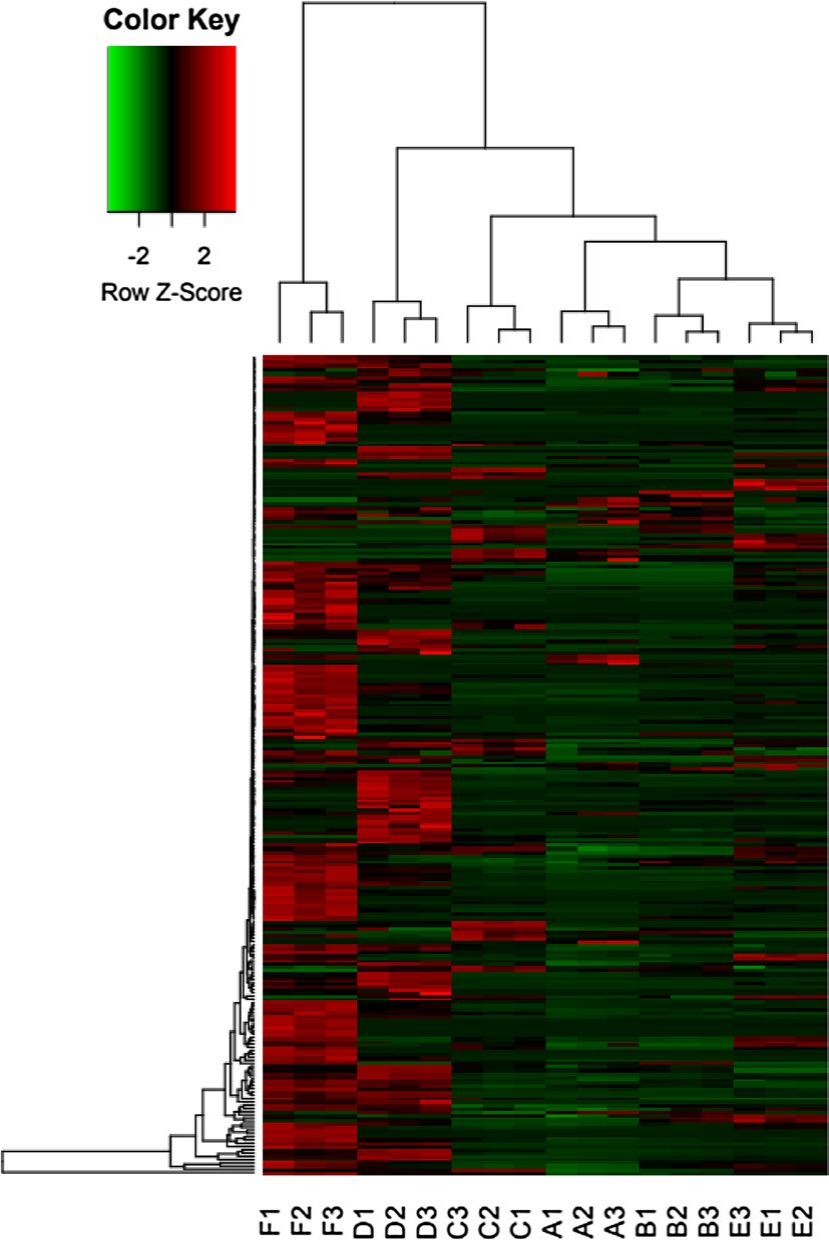
Hierarchical cluster analysis: heat-map reflecting the differences between compounds revealed in the (SPME) GC–MS analysis of volatiles compounds. Capital letters indicates the sample and the number the analytical repetition. Heat-map was generated using R 3.5.2. software^30^.

Looking at the volatile profiles, analysing the six samples, 255 compounds were detected (Table S3), belonging to the following groups: alcohols, aldehydes, anidrides, aromatics, carboxylic acid, esters, eters, furanoids, hydrocarbons, ketones, nitrogenates, sulphurated, terpenes and other (Table 2). Data analysis showed that honeys A, B, C, and E were very similar while D and F were richer in volatile compounds and presented a high dissimilarity from the other samples. Honeys D and F were the most interesting in terms of volatile fingerprints, showing the highest quantity in terpenes and terpenoids (both around 45 ppb compared to the other honeys, ranging from 13 to 15 ppb). Honeys D and F also had the highest amount of carboxylic acids (Table 2). The single compounds were then analysed to identify the compounds significantly different in each sample (Fig. 8).

**Table 2:** Total volatiles compounds divided for chemical group. Data are expressed in ppb.

Sample	Alcohols (ppb)	Aldehydes (ppb)	Anidrides (ppb)	Aromatics (ppb)	Carboxylic acid (ppb)	Esters (ppb)	Eters (ppb)	Furanoids (ppb)	Hydrocarbons (ppb)	Ketones (ppb)	Nitrogenates (ppb)	Sulfurated (ppb)	Terpenes (ppb)
A	7.77	10.90	0.00	0.82	19.72	1.01	0.11	0.25	1.12	2.50	0.03	0.77	13.47
B	8.88	39.85	0.04	0.68	34.77	1.60	0.14	3.93	1.21	3.68	0.13	0.53	15.72
C	31.59	13.10	0.03	1.10	54.65	2.53	0.16	0.66	2.12	5.93	0.11	0.74	13.04
D	27.86	37.30	0.29	9.49	147.43	7.20	0.62	4.10	4.13	14.43	1.59	2.13	45.48
E	20.41	45.24	0.07	1.32	50.22	1.47	0.07	6.59	3.12	6.40	0.30	0.86	13.95
F	29.75	91.30	0.00	7.50	190.71	6.90	0.41	8.25	5.32	33.40	6.57	3.90	46.77

Carboxylic acids are responsible of different scents, depending on carbon chain length. Short chain acids such as acetic acid have for example spicy flavours, while butanoic acid and hexanoic acids determine a rancid aroma^18^. Honey F was the one containing the highest quantity of acetic acid (81.89 ± 4.069 ppb) and the one with a consistent presence of chestnut pollen. Honey F was described having the peculiarly spiced flavour of chestnut together with the vegetable/fruity flavour of wildflower and the bitterness of linden tree and chestnut. Honey F was also the richest in nitrogenated compounds (Fig. 8) such as 2-Methylfuran, detected only in honey F. Hydrate furanoids such as 2-Methyltetrahydro-3-furanone have already been found in chestnut honeys of different countries^71^. Shikimate pathway derivatives such as 2,4-Di-tert-butylphenol, 3-Methylacetophenone and 3,4-Dimethylacetophenone, and some specific monoterpenes such as z-Rose oxide, α-terpinene and cymene, in higher quantities in honey F, were previously associated to linden honey or in general to the genus *Tilia*^71^, that includes different species generally called linden trees for the European species. These species contribute to the production of linden tree honey, which has a taste which is sweet, bitter, medicinal, floral, woody and hay-like^72^. Honey F is the one produced at the lower station, where the linden and chestnut botanical belt starts, and is more definable organoleptically as a mix of *Tilia* and *Castanea* honey than a mountain wildflower honey. Honey F is the only one in which *Tilia* pollen was detected. *Tilia* was not detected by the floristic relevé but is present in the production area of honey F; this could mean that bees are willing to travel far to find this essence. 1-Phenylethanol, 2-Phenylethanol, 2-aminoacetophenone and acetophenone content distinguished honey F with a high content of chestnut pollen coherently with a previous report^73^. Considered as a product related to the shikimic acid pathway as well^74^, acetophenone is formed during phenylpropane metabolism by enzymatic reactions from hydroxy-substituted aromatic acids^75^. According to^73^, 1-Phenylethanol can be associated to a floral odour and may contribute to the characteristic floral aroma of chestnut honey in synergy with other compounds, such as phenylacetaldehyde or 2-phenylethanol. 2-aminoacetophenone is probably not issued by the shikimate pathway but might be the result of tryptophane degradation^76^. In this study tryptophane was not detected by GC–MS but by HPLC Orbitrap. 2-aminoacetophenone is a strong scent^73^ that can also contribute to the flavour of chestnut honey. Moreover, honey F had the highest concentrations of products produced by the non-enzymatic browning of sugars such as 2-acetylfuran, 3-Methylfuran, 2-Ethylfuran, menthofuran, 4,7-Dimethyl-benzofuran and 2,3-Dihydro-benzofuran. Only 4,5-Dimethyl-2-formylfuran was highest in honey E. These results fit in with the large amounts of furan and methylfuran previously measured in^77^ with a headspace dynamic method in the same origin. These furan derivatives cannot be considered specific floral markers because they are associated to specific thermal treatment and storage conditions^77^ but they can be useful to authenticate honey of chestnut origin that is characterized by a bitter, sweet, burnt caramel and woody flavour^78^. Considering instead benzene derivatives, benzoic acid is considered a marker of nectar from the genera *Erica* and *Calluna* and was found predominantly in honey F (besides honey D, 10 ppb, against less than 1 ppb in the other honeys). Honey F, as well as being chestnut pollen prevalent, it is also the only honey with a dominant presence of *Ericaceae* pollen. Nectar from the genera *Erica* and *Calluna* contribute to the production of heather honey^79^, its flavour being characterized by sweet and candy-like notes^80^. Benzaldehyde was also found in a quantity higher than in the other honeys in honey F (29.16 ± 6.36 ppb) and has been previously described as a chestnut honey marker^71^, while in honeys B, D and E it was around 10 ppb and in the remaining less than 2 ppb.

Different compounds that were responsible for the dissimilar profile in honey D were a category that are considered both “non-specific” (deriving from the degradation of carotenoid precursors producing different C9- norisoprenoids) and markers of some specific essences such as *Ericaeae* (heather honey) and were coherently found in higher quantities also in honey F (Fig. 8). For example, significantly higher quantities of α-Isophorone and 4-Oxoisophorone were found only in honeys D and F, while in the other samples they were present only in traces. Other non-specific compounds found in higher quantities in honeys D and F were numerous carboxylic acids (Hexa-2,4-Dienoic acid isomer, Hexanoic acid, Butanoic acid and Acetic acid) and the terpenes Linalool, trans-Linalool oxide and cis-Linalool oxide, Lilac Aldheide and its isomers. These kinds of compound are among the most common in honey, and many VOCs considered nonspecific such as linalool and linalool oxide have been seen to be involved in communication between flowers of fruit crops and their pollinators^81^. Moreover, honeys D and F were the ones containing higher quantities of Hotrienol, a regular monoterpene derived from geranyl pyrophosphate (GPP), among the most common honey terpenes. β-Damascenone, defined as a Rhododendron honey and Ericacee in general honey marker^71,75,82^ was found in a higher quantity in honey D compared to the other honeys (2.49 ppb while in the other honeys it was always lower than 1 ppb) (Fig. 8).

A significant amount of nicotinaldehyde was found in honeys B (6.65 ppb), D (2.6 ppb), E (7.88) and F (3.01 ppb), while this compound was not detected in honeys A (almost pure rhododendron) and C where it was found in lower quantities (0.16 ppb) (Table S3). This is remarkably coherent with the melissopalynological analysis where *Rubus* pollen was found mainly in honeys B (67.12%), D (34.74%), E (44.25%) and F (38.59%), while it was found in far lower amounts in honeys A (11.83%) and C (17.24%). The high correlation between nicotinaldehyde content and percentage of *Rubus* pollen (r = 0.853, *p* < 0.05) qualifies this molecule as a promising marker for *Rubus* pollen occurrence. A lower content in nicotinaldehyde (0.16 ppb) was found in sample C that was characterized by 17.24% of *Rubus* pollen, whereas it was not detected in sample A that was characterized by 11% of *Rubus* pollen. Therefore, cut-off values for nicotinaldehyde as a *Rubus* pollen marker can preliminarily be set at 17%. Raspberry honey is quite rare and only few references exist concerning volatile compounds of this honey produced in Estonia and Slovakia^71^, although without mention of the compound nicotinaldehyde. However, nicotinaldehyde was reported in the chemical composition of volatiles released by the flowers and fruits of the red raspberry (*Rubus idaeus*) by^83^. Nicotinaldehydes could be involved in the synthesis of quinolones, the class of naturally occurring compounds that was abundantly present in samples according to our HPLC Orbitrap analysis. It is clear that there is a need for further studies to ascertain the existence of a common volatile profile for this type of honey^71^. Quinoline alkaloids, sought by bees in both chestnut and raspberry, could therefore have an important role in insect-plant communication. It is important to consider the volatile composition of honey as these substances are the main factors responsible for aroma which, together with other factors such as taste and physical factors, contribute to flavour^17^. Nevertheless, the volatile fingerprint of honey as a recognition method for floral origin is a debated topic because it is rather difficult to find reliable chemical markers for the discrimination of honey collected from different floral sources. Therefore, scientific publications may report different floral markers for honey of the same floral origin.

The organoleptic properties of honey (flavour, colour, aroma, and texture) are important factors in consumer’s choice of together with the new concept of functional food. Some kinds of honey can have higher market values because of peculiar sensorial properties or for the content in compounds with antioxidant and antimicrobial activity^67,84,85^. Therefore, the characterisation of different honeys could increase their commercial value. Mountain wildflower honeys, for example, can be considered particularly valuable, as they are the expression of meadows and pastures biodiversity. In fact, they are included in the presidia to protect following the rules of the Slow Food Foundation for Biodiversity^10^. When transhumance was widespread and regular in the Alps, bees (wild and domesticated) also benefited: the pastures were cared for and cleaned to the benefit not only of the animals, but also of the vegetation, and then the bees^10^.

As exposed, the chemical composition and the consequent properties of honey depend largely on its floral source which is very dependent on the geographical origin of honey^14^. As well, potential contamination sources can characterise different honey production areas^86^, also in the case of organic honey^87^. The production of honey in uncontaminated areas as mountain areas, can be a way of promoting this product so difficult to obtain, together with the promotion of bioactive properties of a sustainable product from marginal territories.

Nevertheless, the honey of the study area, although produced in a limited territory, produced very different honeys according to the different apiary stations and, most importantly, the honeys were not totally definable as wildflower. This highlights the importance of formal analysis (at least melissopalynological analysis if phytochemical is not possible) to characterize honey, that could often reveal food products even more interesting than expected. A better definition of honeys, in addition, could give important cues for beekeeping management, as there are certain key species or plant groups that are particularly important^2^ and a valuable area of further research is to discover why these particular species are important and how landscapes affect bee foraging in different environments. An understanding of the reasons why honeybees target certain plants could help to provide guidance on what constitutes a balanced honeybee diet^2^. The characterization of honeys, furthermore, could lead to the discovery of important compounds both for bees, in particular considering bee behaviour and bee-plant communication, and for human nutrition.

## Conclusion

Our study highlights the difficulty of producing a mountain wildflower honey in the Alps taking into consideration expected aroma and flavour characteristics. This could be attributed to environmental and behavioural features, also linked to the presence of particular neuroactive compounds in some essences. Thorough characterization of the product is essential in order to reveal unexpected characteristics also from a nutraceutical point of view. It is to be hoped that this work will contribute to the valorisation of wildflower honey, this important product of the Alps, and give new impetus to its production conditional on bee behaviour which in turn depends on the anthropic and climatic changes affecting mountain territories.

## Supplementary Information

Supplementary Information.
