# Supplementary material for: Multidisciplinary analysis of Italian Alpine wildflower honey reveals criticalities, diversity and value
Source: Sci Rep. 2021 Sep 29;11:19316. doi: 10.1038/s41598-021-98876-y (PMC8481395; doi:10.1038/s41598-021-98876-y)

SUPPLEMENTARY MATERIALS

Table S1. Table of relevés. Cover indices refer to the Braun-Blanquet abundance/dominance scale: r, rare; +, <1%; 1, 1–5%; 2, 6–25%; 3, 26–50%; 4, 51–75%; 5, 76–100%.

**Relevé code**	**A1**	**A2**	**A3**	**A4**	**B1**	**B2**	**B3**	**B4**	**C1**	**C2**	**C3**	**C4**	**D1**	**D2**	**D3**	**D4**	**E1**	**E2**	**E3**	**E4**	**F1**	**F2**	**F3**	**F4**	
Study area	A	A	A	A	B	B	B	B	C	C	C	C	D	D	D	D	E	E	E	E	F	F	F	F	
Surface (m²)	25	25	25	25	25	25	25	25	25	25	25	25	25	25	25	25	25	25	25	25	25	25	25	25	
Inclination (°)	10	0	6	8	8	7	8	6	16	14	10	14	9	8	13	12	0	40	0	16	36	38	36	32	
Exposure (°)	51	-	70	120	170	165	167	173	184	180	193	185	148	130	136	180	-	350	-	352	225	225	225	225	
Elevation (m a.s.l.)	1685	1683	1681	1683	1073	1072	1073	1075	1624	1625	1627	1626	1748	1750	1752	1749	1805	1802	1805	1807	850	855	850	847	
Trees coverage (%)	1	1	0	1	0	1	1	0	0	30	60	30	0	0	0	0	0	20	10	70	50	50	80	50	
Shrubs coverage (%)	1	1	1	1	0	0	0	0	0	30	55	10	0	0	1	1	0	5	0	1	5	5	20	5	
Grass coverage (%)	98	99	99	98	99	99	99	99	100	70	40	90	100	100	99	99	100	95	100	100	95	95	80	95	
Roks/debris (%)	1	0	0	1	0	0	0	0	0	1	5	0	0	0	0	0	0	0	0	0	0	0	0	0	
Number of species	30	25	27	27	23	26	22	22	37	37	33	39	33	26	35	38	37	21	29	42	26	25	31	34	Pres. (%)
Anthoxanthum odoratum L.	1	+	+	+	+	+	+	+	+	+	.	+	+	+	.	+	+	.	r	1	1	+	+	+	88
Achillea millefolium L.	1	.	+	1	+	1	+	2	2	2	.	2	1	3	2	3	2	+	2	+	+	.	.	1	83
Trifolium repens L.	1	.	+	+	+	+	+	1	+	+	+	1	+	1	+	+	1	.	+	.	+	.	.	+	79
Dactylis glomerata L.	.	.	.	.	1	+	+	+	+	r	.	+	1	+	2	1	+	+	+	.	+	+	.	+	71
Taraxacum officinale Weber	.	+	+	+	1	+	+	2	+	.	.	.	r	.	.	.	2	+	2	+	+	+	+	1	71
Alchemilla vulgaris L. s.s.	+	.	.	+	.	.	.	.	+	1	+	+	r	.	r	+	3	+	1	+	.	r	.	r	63
Festuca nigrescens Lam.	2	1	1	2	.	.	.	.	2	2	+	2	+	1	+	+	+	.	+	1	.	.	.	.	63
Ranunculus acris L.	+	+	+	+	+	+	.	+	1	+	+	+	.	.	.	.	+	.	+	+	r	.	.	.	63
Rumex acetosa L.	+	.	.	.	1	+	+	1	+	.	.	.	1	+	+	+	r	.	1	+	+	.	.	+	63
Phleum alpinum L.	2	+	1	+	.	.	.	.	+	+	.	+	1	+	+	+	r	.	+	1	.	.	.	.	58
Chaerophyllum hirsutum L.	+	.	.	.	.	.	.	.	2	2	1	2	2	r	2	2	+	1	r	+	.	.	.	.	54
Larix decidua Mill.	+	+	+	+	.	.	.	.	.	+	+	1	.	.	r	+	+	+	1	3	.	.	.	.	54
Trifolium pratense L. subsp. *nivale* (Sieber) Asch. et Gr.	+	.	.	+	.	.	.	.	1	+	.	1	2	1	1	1	1	+	1	+	.	.	.	.	54
Poa trivialis L.	.	+	.	.	+	+	+	+	.	.	.	.	+	+	+	.	.	.	+	.	+	+	.	+	50
Veronica chamaedrys L.	+	+	.	+	.	.	.	.	.	.	r	.	+	1	+	1	+	+	+	+	.	.	.	.	50
Deschampsia cespitosa (L.) P. Beauv.	1	4	4	3	.	.	.	.	1	.	.	1	+	.	.	1	+	.	+	+	.	.	.	.	46
Ranunculus montanus Willd.	1	+	+	+	.	.	.	.	+	.	.	.	+	.	+	+	+	.	+	+	.	.	.	.	46
Lotus alpinus (Ser.) Schelich. ex Ramond	+	.	+	.	.	.	.	.	+	+	.	+	.	1	+	+	+	.	+	.	.	.	.	.	42
Rhinanthus alectorolophus (Scop.) Pollich	.	.	.	.	2	2	+	+	.	.	.	.	1	2	+	+	.	.	.	.	r	.	.	+	42
Arrhenatherum elatius (L.) P. Beauv. Ex J. Presl & C. Presl	.	.	.	.	3	3	5	2	r	.	.	.	.	.	.	.	.	.	.	.	3	1	1	1	38
Brachypodium rupestre (Host) Roem. et Shult	.	.	.	.	.	.	.	.	+	+	+	1	.	.	.	.	.	.	.	1	+	4	1	2	38
Potentilla erecta (L.) Raeusch.	+	1	1	+	.	.	.	.	+	r	+	+	.	.	.	.	.	.	.	1	.	.	.	.	38
Fragaria vesca L.	.	.	.	.	.	.	.	.	+	+	+	+	.	.	.	.	r	.	+	.	.	+	2	.	33
Geranium sylvaticum L.	.	.	.	.	+	.	.	r	.	.	r	.	+	.	.	+	+	1	+	.	.	.	.	.	33
Silene vulgaris (Moench) Garcke	.	.	.	.	+	+	+	+	r	.	.	.	+	1	.	.	.	.	.	.	.	.	+	.	33
Myosotis alpestris F.W. Schmidt	.	.	.	.	.	.	.	.	.	.	.	.	+	+	r	r	r	.	2	+	.	.	.	.	29
Phyteuma betonicifolium Vill.	.	.	.	.	.	.	.	.	+	+	.	1	1	+	+	+	.	.	.	.	.	.	.	.	29
Picea abies (L.) H. Karst.	.	.	.	.	.	.	r	.	.	2	4	1	.	.	.	.	.	+	1	1	.	.	.	.	29
Poa alpina L.	.	r	+	.	.	.	.	.	.	.	.	.	+	+	.	+	+	.	+	.	.	.	.	.	29
Rubus idaeus L.	r	.	+	r	.	.	.	.	.	.	1	+	.	.	.	.	.	1	.	+	.	.	.	.	29
Scorzoneroides helvetica (Mérat emend. Widder) Holub	+	.	r	+	.	.	.	.	.	.	.	.	r	r	.	+	.	.	.	r	.	.	.	.	29
Trifolium pratense L. subsp. *pratense*	.	.	.	.	1	2	+	1	.	.	.	.	.	.	.	.	.	.	.	.	+	+	.	+	29
Agrostis capillaris L.	.	.	.	.	+	+	+	+	.	.	.	.	1	+	.	.	.	.	.	.	.	.	.	.	25

Briza media L.	.	.	.	.	.	.	.	.	r	.	.	.	.	.	.	.	r	.	.	.	+	+	+	+	25
Luzula sudetica (Willd.) Schult.	+	+	+	.	.	.	.	.	+	.	.	+	.	.	+	.	.	.	.	.	.	.	.	.	25
Nardus stricta L.	1	+	+	+	.	.	.	.	.	+	.	r	.	.	.	.	.	.	.	.	.	.	.	.	25
Plantago lanceolata L.	.	.	.	.	+	.	.	+	.	.	.	.	.	.	.	.	.	.	.	.	+	r	r	+	25
Senecio ovatus (P. Gaertn., B. Mey. & Scherb.) Willd.	.	+	+	r	.	.	.	.	.	.	+	.	.	.	.	.	.	+	.	1	.	.	.	.	25
Vicia spp.	.	.	.	.	.	.	.	.	.	.	.	.	.	.	r	r	.	.	.	.	+	1	+	+	25
Ajuga pyramidalis L.	.	.	.	.	.	.	.	.	+	+	+	+	.	.	.	.	+	.	.	.	.	.	.	.	21
Bistorta officinalis Delarbre	.	.	.	.	.	.	.	.	.	.	.	.	1	r	+	1	.	.	.	r	.	.	.	.	21
Carex pallescens L.	+	+	+	+	.	.	.	.	.	.	.	.	.	.	+	.	.	.	.	.	.	.	.	.	21
Carum carvi L.	.	.	.	.	.	.	.	.	+	.	.	.	+	+	.	+	+	.	.	.	.	.	.	.	21
Dactylorhiza maculata (L.) Soo	.	.	.	.	.	.	.	.	r	+	+	+	.	.	.	.	+	.	.	.	.	.	.	.	21
Festuca rubra L.	.	.	.	.	.	.	.	.	.	.	.	.	.	.	+	+	.	.	.	.	r	+	.	+	21
Galium anisophyllum Vill.	+	+	+	+	.	.	.	.	.	.	.	.	.	.	+	.	.	.	.	.	.	.	.	.	21
Hieracium murorum L.	.	.	.	.	.	.	.	.	.	.	+	+	.	.	.	.	r	.	r	+	.	.	.	.	21
Imperatoria ostruthium L.	.	.	.	.	.	.	.	.	+	.	.	+	.	.	.	+	.	+	.	r	.	.	.	.	21
Leucanthemum vulgare Lam.	.	.	.	.	.	r	.	.	.	.	.	.	r	2	1	.	r	.	.	.	.	.	.	.	21
Rhododendron ferrugineum L.	+	+	.	+	.	.	.	.	.	.	+	.	.	.	.	.	.	.	.	r	.	.	.	.	21
Veronica officinalis L.	r	.	r	.	.	.	.	.	.	+	.	.	.	.	.	.	+	.	+	.	.	.	.	.	21
Viola tricolor L.	.	.	.	.	.	.	.	.	.	.	.	.	r	+	+	+	r	.	.	.	.	.	.	.	21
Alnus viridis (Chaix) DC.	.	.	.	.	.	.	.	.	.	r	+	2	.	.	.	.	.	+	.	.	.	.	.	.	17
Bellardichloa variegata (Lam.) Kerguelen	1	+	+	1	.	.	.	.	.	.	.	.	.	.	.	.	.	.	.	.	.	.	.	.	17
Cerastium holosteoides Fr.	.	.	.	.	.	.	.	.	.	.	.	.	.	+	+	+	+	.	.	.	.	.	.	.	17
Heracleum sphondylium L.	.	.	.	.	1	1	+	1	.	.	.	.	.	.	.	.	.	.	.	.	.	.	.	.	17
Lotus corniculatus L.	.	.	.	.	.	+	.	+	.	.	.	.	+	.	.	.	.	.	.	.	+	.	.	.	17
Luzula nivea (L.) Lam. et DC.	.	.	r	.	.	.	.	.	.	r	+	1	.	.	.	.	.	.	.	.	.	.	.	.	17
Pimpinella major (L.) Huds.	.	.	.	.	+	1	+	+	.	.	.	.	.	.	.	.	.	.	.	.	.	.	.	.	17
Plantago media L.	.	.	.	.	.	.	.	.	+	.	.	.	.	.	+	.	+	.	+	.	.	.	.	.	17
Polygala alpestris Rchb.	.	.	.	.	.	.	.	.	+	+	.	r	.	.	+	.	.	.	.	.	.	.	.	.	17
Prunella grandiflora (L.) Scholler	.	.	.	.	.	.	.	.	+	+	+	+	.	.	.	.	.	.	.	.	.	.	.	.	17
Robinia pseudoacacia L.	.	.	.	.	.	.	.	.	.	.	.	.	.	.	.	.	.	.	.	.	+	+	1	1	17
Salvia pratensis L.	.	.	.	.	+	1	+	1	.	.	.	.	.	.	.	.	.	.	.	.	.	.	.	.	17
Thymus pseudochamaedrys (Heinr. Braun) Ronniger	+	.	.	.	.	.	.	.	+	+	.	+	.	.	.	.	.	.	.	.	.	.	.	.	17
Malus domestica Borkh.	.	.	.	.	.	.	.	.	.	.	.	.	.	.	.	.	.	.	.	.	3	2	.	1	13
Cynosurus cristatus L.	.	.	.	.	.	.	.	.	r	.	.	.	.	.	.	.	.	.	.	.	1	.	.	+	13
Arnica montana L.	.	r	.	.	.	.	.	.	.	r	+	.	.	.	.	.	.	.	.	.	.	.	.	.	13
Athyrium filix-femina(L.) Roth	.	.	.	.	.	.	.	.	.	+	+	+	.	.	.	.	.	.	.	.	.	.	.	.	13
Carduus defloratus L.	.	.	.	.	.	.	.	.	+	.	r	.	.	.	.	.	r	.	.	.	.	.	.	.	13
Carex panicea L.	.	1	+	+	.	.	.	.	.	.	.	.	.	.	.	.	.	.	.	.	.	.	.	.	13
Carex pilulifera L.	+	.	.	+	.	.	.	.	.	.	+	.	.	.	.	.	.	.	.	.	.	.	.	.	13
Cirsium erisithales (Jacq.) Scop.	.	.	.	.	.	.	.	.	.	+	1	+	.	.	.	.	.	.	.	.	.	.	.	.	13
Elymus repens (L.) Gould	.	.	.	.	+	+	+	.	.	.	.	.	.	.	.	.	.	.	.	.	.	.	.	.	13
Epilobium angustifolium L.	.	.	r	.	.	.	.	.	.	.	.	.	.	.	.	.	r	5	.	.	.	.	.	.	13
Fraxinus excelsior L.	.	.	.	.	.	+	r	.	.	.	.	.	.	.	.	.	.	.	.	.	.	.	+	.	13
*Galium album*Mill.	.	.	.	.	.	.	.	.	.	.	.	.	.	.	.	.	.	.	.	.	+	.	+	+	13
Glechoma hederacea L.	.	.	.	.	.	.	.	.	.	.	.	.	.	.	.	.	.	.	.	.	.	r	+	1	13
Holcus lanatus L.	.	.	.	.	.	.	.	.	.	.	.	.	.	.	.	.	.	.	.	.	+	r	.	+	13
Juniperus communis L.	.	.	.	.	.	.	.	.	.	1	+	+	.	.	.	.	.	.	.	.	.	.	.	.	13
Knautia drymeia Heuff.	.	.	.	.	.	.	.	.	+	+	.	+	.	.	.	.	.	.	.	.	.	.	.	.	13
Lathyrus pratensis L.	.	.	.	.	.	.	.	.	.	.	.	.	+	.	.	.	.	.	.	.	+	+	.	.	13
Leontodon hispidus L.	.	.	.	.	.	.	.	.	.	.	.	.	.	.	.	.	.	.	.	.	2	+	.	+	13
Lolium perenne L.	.	.	.	.	.	.	.	1	.	.	.	.	+	.	.	.	.	.	.	.	.	.	.	+	13
Phleum pratense L.	.	.	.	.	.	+	.	.	.	.	.	.	.	.	+	+	.	.	.	.	.	.	.	.	13
Poa annua L.	.	.	r	.	.	.	.	.	.	.	.	.	.	.	.	.	+	.	+	.	.	.	.	.	13

Prunus avium (L.) L.	.	.	.	.	.	.	.	.	.	.	.	.	.	.	.	.	.	.	.	.	.	2	+	1	13
Pteridium aquilinum (L.) Kuhn	.	.	.	.	.	.	.	.	2	.	+	.	.	.	.	.	.	.	.	2	.	.	.	.	13
Ranunculus villarsii DC.	.	.	.	.	.	.	.	.	.	.	.	.	+	.	+	1	.	.	.	.	.	.	.	.	13
Schedonorus pratensis (Huds.) P. Beauv.	.	.	.	.	1	+	.	+	.	.	.	.	.	.	.	.	.	.	.	.	.	.	.	.	13
Sorbus aucuparia L.	.	.	.	.	.	.	.	.	.	.	.	+	.	.	.	.	.	+	.	+	.	.	.	.	13
Trisetaria flavescens (L.) Baumg.	.	.	.	.	.	.	.	.	.	.	.	.	+	.	.	.	.	.	.	.	+	.	.	+	13
Trollius europaeus L.	.	.	.	.	.	.	.	.	.	.	.	.	.	.	+	+	.	.	.	r	.	.	.	.	13
Urtica dioica L.	.	.	.	.	.	.	+	.	.	.	.	.	.	.	.	.	.	1	.	.	.	.	.	+	13
Vicia sepium L.	.	.	.	.	+	+	+	.	.	.	.	.	.	.	.	.	.	.	.	.	.	.	.	.	13
Agrimonia eupatoria L.	.	.	.	.	.	.	.	.	.	.	.	.	.	.	.	.	.	.	.	.	.	.	r	+	8
Campanula scheuchzeri Vill.	.	.	.	.	.	.	r	.	.	r	.	.	.	.	.	.	.	.	.	.	.	.	.	.	8
Centaurea nervosa Willd.	.	.	.	.	.	.	.	.	.	.	.	.	r	.	+	.	.	.	.	.	.	.	.	.	8
Clinopodium vulgare L.	.	.	.	.	.	.	.	.	.	.	.	.	.	.	.	.	.	.	.	.	.	r	.	+	8
Crepis vesicaria L.	.	.	.	.	.	+	+	.	.	.	.	.	.	.	.	.	.	.	.	.	.	.	.	.	8
Erica carnea L.	.	.	.	.	.	.	.	.	.	2	+	.	.	.	.	.	.	.	.	.	.	.	.	.	8
Euphrasia spp.	.	.	.	.	.	.	.	.	+	.	.	r	.	.	.	.	.	.	.	.	.	.	.	.	8
Festuca heterophylla Lam.	.	.	.	.	.	.	.	.	.	.	.	.	.	.	.	.	.	.	.	.	.	+	2	.	8
Hypericum maculatum Crantz	.	.	.	.	.	.	.	.	+	.	.	1	.	.	.	.	.	.	.	.	.	.	.	.	8
Luzula luzulina (Vill.) Dalla Torre et Sarnth.	.	.	.	.	.	.	.	.	.	.	.	.	.	.	.	.	+	.	.	+	.	.	.	.	8
Luzula sylvatica (Huds.) Gaudin	.	.	.	.	.	.	.	.	.	.	.	.	.	.	.	.	.	+	.	3	.	.	.	.	8
Maianthemum bifolium (L.) F.W. Schmidt	.	.	.	.	.	.	.	.	.	.	+	.	.	.	.	.	.	.	.	1	.	.	.	.	8
Oxalis acetosella L.	.	.	.	.	.	.	.	.	.	.	2	.	.	.	.	.	.	.	.	+	.	.	.	.	8
Petasites albus (L.) Gaertn.	.	.	.	.	.	.	.	.	.	.	.	.	.	.	.	.	.	+	.	+	.	.	.	.	8
Pilosella officinarum Vaill.	+	.	.	.	.	.	.	.	.	+	.	.	.	.	.	.	.	.	.	.	.	.	.	.	8
Plantago major L.	.	.	.	.	.	.	.	.	.	.	.	.	.	.	.	.	.	.	1	.	.	.	.	+	8
Potentilla aurea L.	+	+	.	.	.	.	.	.	.	.	.	.	.	.	.	.	.	.	.	.	.	.	.	.	8
Rosa spp.	.	.	.	.	.	.	.	.	.	.	.	.	.	.	.	.	.	.	.	.	.	+	+	.	8
Rumex alpinus L.	.	.	.	.	.	.	.	.	.	.	.	.	.	.	.	.	.	+	+	.	.	.	.	.	8
Rumex scutatus L.	.	.	.	.	.	.	.	.	.	+	.	.	.	.	.	r	.	.	.	.	.	.	.	.	8
Salix caprea L.	.	.	.	.	.	.	.	.	.	.	.	+	.	.	.	.	.	+	.	.	.	.	.	.	8
Sanguisorba minor Scop.	.	.	.	.	.	.	.	.	.	.	.	.	.	.	.	.	.	.	.	.	.	r	+	.	8
Silene dioica (L.) Clairv.	.	.	.	.	.	.	.	.	.	.	.	.	.	+	.	.	.	.	.	r	.	.	.	.	8
Silene nutans L.	.	.	.	.	.	.	.	.	.	+	.	.	.	.	.	+	.	.	.	.	.	.	.	.	8
Stellaria palustris Retz.	.	.	+	+	.	.	.	.	.	.	.	.	.	.	.	.	.	.	.	.	.	.	.	.	8
Vaccinium myrtillus L.	.	.	.	.	.	.	.	.	.	.	2	.	.	.	.	.	.	.	.	+	.	.	.	.	8
Vaccinium vitis-idaea L.	.	r	.	r	.	.	.	.	.	.	.	.	.	.	.	.	.	.	.	.	.	.	.	.	8
Veratrum album L.	.	.	.	.	.	.	.	.	.	.	.	.	.	.	1	1	.	.	.	.	.	.	.	.	8
Viola biflora L.	.	.	.	.	.	.	.	.	.	.	1	.	.	.	.	.	.	.	.	+	.	.	.	.	8
Ajuga reptans L.	.	.	.	.	.	.	.	.	.	.	.	.	.	.	.	.	.	.	.	.	.	.	r	.	4
Anthriscus sylvestris (L.) Hoffm.	.	.	.	.	+	.	.	.	.	.	.	.	.	.	.	.	.	.	.	.	.	.	.	.	4
Anthyllis vulneraria L. subsp. *vulneraria*	.	.	.	.	.	.	.	.	.	.	.	.	.	.	+	.	.	.	.	.	.	.	.	.	4
Arabis turrita L.	.	.	.	.	.	.	.	.	.	.	.	.	.	.	.	.	.	.	.	.	.	.	+	.	4
Artemisia vulgaris L.	.	.	.	.	.	.	.	.	.	.	.	.	.	.	.	.	.	.	.	.	.	.	.	r	4
Betula pendula Roth	.	.	.	.	.	.	.	.	.	.	.	+	.	.	.	.	.	.	.	.	.	.	.	.	4
Calluna vulgaris (L.) Hull	.	.	.	.	.	.	.	.	.	+	.	.	.	.	.	.	.	.	.	.	.	.	.	.	4
Campanula barbata L.	.	.	.	.	.	.	.	.	.	.	.	.	.	r	.	.	.	.	.	.	.	.	.	.	4
*Campanula trachelium*L.	.	.	.	.	.	.	.	.	.	.	.	.	.	.	.	.	.	.	.	.	.	.	r	.	4
Capsella bursa-pastoris (L.) Medik.	.	.	.	.	.	.	.	+	.	.	.	.	.	.	.	.	.	.	.	.	.	.	.	.	4
Cardamine bellidifolia L.	.	.	.	r	.	.	.	.	.	.	.	.	.	.	.	.	.	.	.	.	.	.	.	.	4
Carex nigra (L.) Reichard	.	+	.	.	.	.	.	.	.	.	.	.	.	.	.	.	.	.	.	.	.	.	.	.	4
Castanea sativa Mill.	.	.	.	.	.	.	.	.	.	.	.	.	.	.	.	.	.	.	.	.	.	.	.	1	4
Centaurea jacea L.	.	.	.	.	+	.	.	.	.	.	.	.	.	.	.	.	.	.	.	.	.	.	.	.	4
Chenopodium album L.	.	.	.	.	.	.	.	.	.	.	.	.	.	.	.	.	.	.	.	.	.	.	.	r	4

Cirsium palustre (L.) Scop.	.	.	.	.	.	.	.	.	.	.	.	.	.	.	.	r	.	.	.	.	.	.	.	.	4
Clematis vitalba L.	.	.	.	.	.	.	.	.	.	.	.	.	.	.	.	.	.	.	.	.	.	.	r	.	4
*Corylus avellana*L.	.	.	.	.	.	.	.	.	.	.	.	.	.	.	.	.	.	.	.	.	.	.	3	.	4
Crocus vernus (L.) Hill	.	.	.	.	.	.	.	.	.	.	.	.	.	.	.	.	.	.	.	r	.	.	.	.	4
Dryopteris filix-mas (L.) Schott	.	.	.	.	.	.	.	.	.	.	.	.	.	.	.	.	.	.	.	+	.	.	.	.	4
Euonymus europaeus L.	.	.	.	.	.	.	.	.	.	.	.	.	.	.	.	.	.	.	.	.	.	.	+	.	4
Festuca ovina L.	.	.	.	.	.	.	.	.	.	.	.	.	.	.	.	+	.	.	.	.	.	.	.	.	4
Festuca scabriculmis (Hack.) K. Richt.	.	.	.	.	.	.	.	.	.	.	.	.	.	.	.	.	.	.	.	r	.	.	.	.	4
Ficus carica L.	.	.	.	.	.	.	.	.	.	.	.	.	.	.	.	.	.	.	.	.	.	.	+	.	4
Galium spp.	.	.	.	.	.	+	.	.	.	.	.	.	.	.	.	.	.	.	.	.	.	.	.	.	4
Galium uliginosum L.	.	r	.	.	.	.	.	.	.	.	.	.	.	.	.	.	.	.	.	.	.	.	.	.	4
*Geranium robertianum*L.	.	.	.	.	.	.	.	.	.	.	.	.	.	.	.	.	.	.	.	.	.	.	+	.	4
Geum montanum L.	.	.	.	.	.	.	.	.	.	.	.	.	.	.	.	+	.	.	.	.	.	.	.	.	4
Geum rivale L.	.	.	.	.	.	.	.	.	.	.	.	.	.	.	.	.	r	.	.	.	.	.	.	.	4
Geum urbanum L.	.	.	.	.	.	.	.	.	.	.	.	.	.	.	.	.	.	.	.	.	.	.	+	.	4
Hederahelix L.	.	.	.	.	.	.	.	.	.	.	.	.	.	.	.	.	.	.	.	.	.	.	+	.	4
Homogyne alpina (L.) Cass.	.	.	.	.	.	.	.	.	.	.	.	.	.	.	.	.	.	.	.	+	.	.	.	.	4
Hypericum perforatum L.	.	.	.	.	.	.	.	.	.	.	.	.	.	.	.	1	.	.	.	.	.	.	.	.	4
Koeleria pyramidata (Lam.) Domin	.	.	.	.	.	.	.	.	.	.	.	.	.	.	.	.	.	.	.	.	.	r	.	.	4
Lamium album L.	.	.	.	.	.	.	.	.	.	.	.	.	.	.	.	.	.	.	.	r	.	.	.	.	4
Medicago sativa L.	.	.	.	.	.	+	.	.	.	.	.	.	.	.	.	.	.	.	.	.	.	.	.	.	4
Plantago alpina L.	.	.	.	.	.	.	.	.	.	.	.	.	.	.	.	.	+	.	.	.	.	.	.	.	4
Platanthera chlorantha (Custer) Rchb.	.	.	.	.	.	.	.	.	.	.	.	.	.	.	.	.	.	.	.	.	.	.	r	.	4
Pseudorchis albida (L.) A. Love et D. Love	r	.	.	.	.	.	.	.	.	.	.	.	.	.	.	.	.	.	.	.	.	.	.	.	4
Pyrus pyraster Burgsd.	.	.	.	.	.	.	.	.	.	.	.	.	.	.	.	.	.	.	.	.	+	.	.	.	4
Rubus ulmifolius Scott	.	.	.	.	.	.	.	.	.	.	.	.	.	.	.	.	.	.	.	.	.	.	.	+	4
Solidago virgaurea L.	.	.	.	.	.	.	.	.	.	.	.	.	.	.	.	.	.	.	.	+	.	.	.	.	4
Sonchus oleraceus L.	.	.	.	.	.	.	.	.	.	.	.	.	.	.	.	.	.	.	.	.	.	.	r	.	4
Trifolium montanum L.	.	.	.	.	.	.	.	.	.	.	.	.	.	.	+	.	.	.	.	.	.	.	.	.	4
Vincetoxicum hirundinaria Medik.	.	.	.	.	.	.	.	.	.	.	.	.	.	.	.	.	.	.	.	.	.	.	+	.	4

Table S2. Relative frequencies of the main pollen types in honeys.

	SAMPLE CODE					
	**A**	**B**	**C**	**D**	**E**	**F**
**Dominant pollen (>45%)**	*Rhododendron* (47.18)	*Rubus* (67.12)	*Rhododendron* (62.93)			*Castanea* (96.4)
**Secondary pollen (16–45%)**	*Pedicularis* (26.47)	*Rhododendron* (21.24)	*Rubus* (17.24)	*Rhododendron* (34.27)<br>*Rubus* (34.74)	*Rhododendron* (34.14)<br>*Rubus* (44.25)	*Ericaceae* (32.45)<br>*Rubus* (38.59)
**Important minor pollen (3–15%)**	*Lotus alpinus* (9.17)<br>*Rubus* (11.83)	*Cruciferae: Cardamine* f. (3.31)	*Lotus alpinus* (3.72)<br>*Trifolium repens* gr. (8.62)	*Anthyllis* f. (14.23)<br>*Campanulaceae: Campanula, Phyteuma* (5.1)<br>*Pedicularis* (4.17)<br>*Potentilla/fragaria* (3.1)	*Lotus alpinus* (5.22)<br>*Trifolium repens* gr. (3.48)<br>*Hippocrepis/Coronilla* (3.13)	*Potentilla/fragaria* (3.47)<br>*Sorbus* f. (6.14)<br>*Tilia* (3.5)<br>*Trifolium repens* gr. (4.38)
**Minor pollen (<3%)**	*Clematis* (2.21)	*Pedicularis* (1.73)<br>*Sorbus* f. (1.75)<br>*Umbelliferae: Anthryscus* f. (1.7)	*Campanulaceae: Campanula, Phyteuma* (1.1)<br>*Pedicularis* (2.1)	*Centaurea* (2.00)<br>*Polygonum bistorta* (2.3)	*Liliaceae: Lilium, Allium, Maianthemum* f. (2.78)<br>*Onobrychis* (2.09)<br>*Potentilla/fragaria* (2.43)	*Clematis* (1.7)<br>*Polygonum bistorta* (1.75)<br>*Campanulaceae: Campanula, Phyteuma* (1.7)
**Sporadic pollen (1% or less)**	*Acer*<br>*Ailanthus*<br>*Aster/Solidago*<br>*Astragalus*<br>*Campanulaceae: Campanula, Phyteuma*<br>*Castanea*<br>*Centaurea*<br>*Chamaerops*<br>*Chenopodium/Amaranthus*<br>*Compositae* S*: Carduus, Cirsium* f.<br>*Dryas* f.<br>*Filipendula*<br>*Gleditsia*<br>*Graminaceae*<br>*Hypericum* f.<br>*Juniperus*<br>*Linaria*<br>*Luzula*<br>honeydew<br>*Pinaceae*<br>*Potentilla/fragaria*<br>*Prunus*<br>*Quercus* r.<br>*Ranuncolaceae: Anemone, Ranunculus* f.<br>*Rosa* f.<br>*Salix*<br>*Saxifraga*<br>*Sedum/Sempervivum*<br>*Stachys f.*<br>*Thalictrum*<br>*Thymus*<br>*Trifolium pratense gr.*<br>*Trifolium repens gr.*	*Astrantia* f.<br>*Campanulaceae: Campanula, Phyteuma*<br>*Castanea*<br>*Compositae* S*: Carduus,Cirsium* f.<br>*Epilobium*<br>*Filipendula*<br>*Graminaceae*<br>*Helianthemum*<br>*Juniperus*<br>*Knautia/Scabiosa*<br>*Liliaceae: Lilium, Allium, Maianthemum* f.<br>*Lonicera*<br>*Lotus alpinus*<br>*Luzula*<br>honeydew<br>*Myosotis*<br>*Plantago*<br>*Polygonum bistorta*<br>*Potentilla/fragaria*<br>*Rhinanthus* f.<br>*Rosa* f.<br>*Rumex*<br>*Salix*<br>*Sambucus*<br>*Sedum/Sempervivum*<br>*Thalictrum*<br>*Trifolium repens* gr.<br>*Vicia*	*Achillea*<br>*Buddleja*<br>*Castanea*<br>*Centaurea*<br>*Compositae* S: *Carduus, Cirsium* f.<br>*Compositae* T: *Taraxacum, Crepis, Hieracium* f.<br>Graminaceae<br>*Helianthemum*<br>*Hippocrepis/Coronilla*<br>*Juniperus*<br>*Knautia/Scabiosa*<br>*Liliaceae: Lilium, Allium, Maianthemum* f.<br>*Luzula*<br>honeydew<br>*Plantago*<br>*Polygonum bistorta*<br>*Potentilla/fragaria*<br>*Prunella* f.<br>*Rumex*<br>*Saxifraga*<br>*Sedum/Sempervivum*<br>*Thymus*<br>*Trifolium pratense* gr.	*Acer*<br>*Actinidia*<br>*Aruncus*<br>*Carex*<br>*Castanea*<br>*Corylaceae*<br>*Fraxinus*<br>*Graminaceae*<br>*Helianthemum*<br>*Juniperus*<br>*Lotus alpinus*<br>*Luzula*<br>*honeydew*<br>*Quercus r.*<br>*Robinia*<br>*Salix*<br>*Saxifraga*<br>*Sedum/Sempervivum*<br>*Sorbus* f.<br>*Thalictrum*	*Allium* f.<br>*Alnus*<br>*Anthyllis* f.<br>*Castanea*<br>*Clematis*<br>*Graminaceae*<br>*Helianthemum*<br>*Juniperus*<br>*Lonicera*<br>*honeydew*<br>*Papaver*<br>*Pinaceae*<br>*Plantago*<br>*Robinia*<br>*Salix*<br>*Salvia* f.<br>*Sambucus*<br>*Sedum/Sempervivum*<br>*Thalictrum*<br>*Umbelliferae: Anthryscus* f.	*Acer*<br>*Actinidia*<br>*Aruncus*<br>*Centaurea*<br>*Crocus*<br>*Fraxinus*<br>*Graminaceae*<br>*Helianthemum*<br>*Juniperus*<br>*Ligustrum* f.<br>*Lonicera*<br>*Luzula*<br>honeydew<br>*Papaver*<br>*Parthenocissus*<br>*Plantago*<br>*Prunus*<br>*Quercus r.*<br>*Saxifraga*<br>*Sedum/Sempervivum*<br>*Thymus*

Table S3. Volatiles compounds detected in honeys (Rt, retention time).

			CODE SAMPLES											
			A		B		C		D		E		F	
COMPOUNDS	CAS NUMBER	Rt	mean	±SD	mean	±SD	mean	±SD	mean	±SD	mean	±SD	mean	±SD
Alcohols														
1-Octanol	111-87-5	2.53	0.037	0.014	0.000	0.000	0.000	0.000	0.110	0.035	0.012	0.002	0.000	0.000
Nonanol	111-84-2	3.23	0.000	0.000	0.001	0.000	0.000	0.000	0.086	0.013	0.033	0.013	0.147	0.066
2-Propanol	67-63-0	3.70	0.034	0.002	0.031	0.005	0.055	0.032	0.115	0.060	0.121	0.014	0.093	0.021
Ethanol	64-17-5	3.80	2.537	0.630	5.635	0.382	20.917	0.245	11.790	0.406	11.613	0.632	9.279	0.566
2-Methyl-3-buten-2-ol	115-18-4	7.35	0.569	0.084	0.214	0.005	1.090	0.120	2.888	2.308	1.260	0.137	0.476	0.089
Isobutanol	78-83-1	10.38	0.066	0.011	0.189	0.018	0.390	0.008	0.143	0.210	0.221	0.031	0.187	0.072
2-Butanol	78-92-2	10.46	0.001	0.000	0.047	0.006	0.000	0.000	0.271	0.227	0.001	0.000	0.040	0.035
3-Pentanol	584-02-1	11.08	0.016	0.004	0.000	0.000	0.001	0.000	0.080	0.044	0.001	0.000	0.083	0.029
1-Methoxy-2-Propanol	107-98-2	11.48	0.000	0.000	0.000	0.000	0.000	0.000	0.000	0.000	0.021	0.006	0.001	0.000
2-Pentanol	6032-29-7	11.69	0.011	0.003	0.000	0.000	0.013	0.002	0.139	0.024	0.001	0.000	0.054	0.004
1-Butanol	71-36-3	12.73	0.048	0.014	0.000	0.000	0.128	0.030	0.249	0.054	0.114	0.020	0.063	0.010
1-Penten-3-ol	616-25-1	13.47	0.028	0.012	0.052	0.016	0.006	0.002	0.062	0.026	0.016	0.004	0.053	0.022
2-Hexanol	626-93-7	13.84	0.007	0.002	0.027	0.005	0.025	0.002	0.029	0.007	0.022	0.004	0.018	0.005
Isoamyl alcohol	123-51-3	15.44	0.232	0.071	0.802	0.062	0.755	0.102	1.938	0.216	0.848	0.067	1.087	0.075
3-Methyl-3-buten-2-ol	763-32-6	16.79	0.378	0.101	0.127	0.031	0.853	0.125	0.205	0.058	2.181	0.143	2.838	0.040
1-Pentanol	71-41-0	16.95	0.001	0.000	0.001	0.000	0.001	0.000	0.004	0.000	0.000	0.000	0.001	0.000
E- 2-Methyl-3-penten-1-ol	62238-37-3	18.57	0.000	0.000	0.000	0.000	0.000	0.000	0.146	0.026	0.007	0.002	0.272	0.025
Prenol	4675-87-0	19.08	0.192	0.045	0.001	0.000	1.015	0.178	0.007	0.001	0.008	0.002	0.043	0.009
2-Heptanol	543-49-7	19.18	0.027	0.007	0.043	0.006	0.389	0.011	0.093	0.018	0.022	0.001	0.104	0.005
2-Butoxyethanol	111-76-2	21.23	0.100	0.012	0.045	0.024	0.001	0.000	0.000	0.000	0.022	0.007	0.000	0.000
2-Cyclopropyl-2-pentanol	24230-08-8	21.68	0.000	0.000	0.148	0.003	0.091	0.012	0.001	0.000	0.000	0.000	0.192	0.036
Hotrienol	20053-88-7	25.95	0.668	0.081	0.967	0.145	0.901	0.186	4.683	0.481	1.200	0.105	3.270	0.456
2-Phenylpropan-2-ol	617-94-7	28.77	0.001	0.000	0.032	0.003	0.006	0.008	0.000	0.000	0.001	0.000	0.001	0.000
1-Phenylethanol	98-85-1	29.86	0.001	0.000	0.001	0.000	0.001	0.000	0.000	0.000	0.001	0.000	6.591	0.580
Benzyl alcohol	100-51-6	31.02	1.798	0.569	0.076	0.005	3.352	0.902	3.826	1.057	0.000	0.000	4.854	1.463
2-Phenylethanol	60-12-8	31.69	1.017	0.963	0.443	0.060	1.596	0.448	0.995	0.129	2.684	0.265	2.324	0.278
Aldehydes														
Ethanal	75-07-0	1.73	0.130	0.087	0.163	0.010	0.479	0.013	0.298	0.034	0.403	0.020	0.387	0.053
Butanal	123-72-8	2.85	0.000	0.000	0.001	0.000	0.001	0.000	0.001	0.000	0.001	0.000	0.015	0.006
2-Methyl-butanal	96-17-3	3.33	0.027	0.003	0.040	0.003	0.021	0.008	0.069	0.014	0.083	0.014	0.576	0.010
3-Methylbutanal	590-86-3	3.42	0.028	0.008	0.070	0.002	0.087	0.017	0.263	0.029	0.127	0.018	1.092	0.074
Pentanal	110-62-3	4.77	0.000	0.000	0.000	0.000	0.000	0.000	0.006	0.001	0.001	0.000	0.001	0.000
Hexanal	66-25-1	8.99	0.060	0.017	0.122	0.029	0.082	0.016	0.082	0.058	0.287	0.033	0.402	0.043
2-Methyl- 2-butenal	1115-11-3	9.32	0.063	0.013	0.129	0.018	0.114	0.015	0.129	0.051	1.385	0.126	0.814	0.118
Heptanal	111-71-7	14.06	0.130	0.048	0.175	0.016	0.084	0.014	0.114	0.028	0.182	0.045	0.309	0.032
3-Methyl-2-butenal	107-86-8	14.44	0.233	0.065	0.260	0.131	0.317	0.046	0.243	0.066	1.172	0.201	0.656	0.094
Octanal	124-13-0	17.97	0.132	0.071	0.302	0.085	0.115	0.021	0.136	0.007	0.318	0.078	0.317	0.037
1-Formylcyclopentene	ID#: 4768	18.28	0.000	0.000	0.057	0.008	0.000	0.000	0.014	0.005	0.094	0.009	0.020	0.006
Nonanal	124-19-6	20.98	2.349	0.521	8.616	1.051	3.878	0.522	7.947	1.798	11.034	1.371	16.436	3.173

Furfural	98-01-1	22.59	1.071	0.302	3.018	0.400	1.528	0.242	6.304	1.751	4.955	0.233	10.830	2.116
Decanal	112-31-2	23.53	0.335	0.096	0.001	0.000	0.000	0.000	0.000	0.000	0.000	0.000	0.000	0.000
Benzaldehyde	100-52-7	23.95	1.754	0.393	9.390	0.943	2.019	0.223	11.003	2.821	8.527	0.314	29.157	6.360
Lilac Aldheide	67920-63-2	24.39	1.842	0.401	4.311	0.138	1.522	0.625	5.931	0.843	3.436	0.135	6.957	0.719
Lilac Aldheide isomer		24.66	1.392	0.259	4.393	0.160	1.654	0.121	0.000	0.000	0.001	0.000	4.975	0.382
Undecanal	112-44-7	25.82	0.009	0.001	0.057	0.015	0.004	0.001	0.103	0.029	0.094	0.022	0.467	0.062
Myrtenal	564-94-3	26.10	0.006	0.001	0.001	0.000	0.001	0.000	0.001	0.000	0.030	0.004	0.000	0.000
2-Phenyl ethanal	122-78-1	26.38	1.289	0.254	1.734	0.125	0.830	0.175	1.679	0.287	4.514	0.383	10.369	1.375
Cumaldheyde	122-03-2	26.64	0.001	0.000	0.000	0.000	0.000	0.000	0.000	0.000	0.000	0.000	0.046	0.004
Salicylaldehyde	90-02-8	27.07	0.010	0.001	0.234	0.051	0.028	0.006	0.167	0.023	0.204	0.027	0.545	0.097
Nicotinaldehyde	500-22-1	27.59	0.000	0.000	6.651	1.061	0.157	0.022	2.597	0.384	7.876	1.310	3.014	0.648
2-(4'-Methylphenyl)-propanal	ID#: 32380	28.29	0.000	0.000	0.001	0.000	0.001	0.000	0.001	0.000	0.000	0.000	2.134	0.516
3-Phenylpropionaldehyde	104-53-0	29.09	0.000	0.000	0.020	0.004	0.000	0.000	0.000	0.000	0.000	0.000	0.392	0.038
2-Caren-10-al	ID WILEY 387175	29.39	0.000	0.000	0.000	0.000	0.036	0.009	0.001	0.000	0.001	0.000	0.082	0.035
2-Phenyl-2-butenal	4411-89-6	31.21	0.000	0.000	0.000	0.000	0.001	0.000	0.000	0.000	0.014	0.006	0.386	0.084
Anisaldehyde	123-11-5	32.67	0.001	0.000	0.033	0.022	0.000	0.000	0.000	0.000	0.000	0.000	0.000	0.000
Cinnamaldehyde	104-55-2	32.81	0.036	0.005	0.065	0.014	0.136	0.032	0.081	0.031	0.464	0.088	0.903	0.255
Lilial aldheide	80-54-6	32.93	0.006	0.001	0.001	0.000	0.000	0.000	0.016	0.002	0.001	0.000	0.001	0.000
5-hydroxymethylfurfural	67-47-0	39.36	0.001	0.000	0.001	0.000	0.001	0.000	0.001	0.000	0.032	0.014	0.001	0.000
Vanillin	121-33-5	40.46	0.000	0.000	0.001	0.000	0.000	0.000	0.115	0.043	0.009	0.004	0.019	0.008
Anidrides														
Glutaconic anhydride	5926-95-4	32.33	0.000	0.001	0.035	0.012	0.025	0.012	0.291	0.056	0.074	0.029	0.000	0.000
Aromatics														
Ethyl-benzene	100-41-4	10.87	0.006	0.000	0.000	0.000	0.001	0.000	0.001	0.000	0.012	0.005	0.011	0.000
p-Xilene	106-42-3	11.55	0.001	0.000	0.000	0.000	0.001	0.000	0.063	0.014	0.028	0.006	0.024	0.000
Pyridin	110-86-1	13.79	0.000	0.000	0.001	0.002	0.000	0.000	0.001	0.000	0.006	0.000	0.012	0.003
2-pentyl-furan	3777-69-3	16.14	0.001	0.000	0.015	0.005	0.011	0.004	0.025	0.008	0.001	0.000	0.045	0.005
1,2-Dihydro-1,1,6-trimethyl-naphthalene	30364-38-6	28.51	0.001	0.000	0.001	0.000	0.001	0.000	0.336	0.032	0.001	0.000	0.001	0.000
Imidazole	288-32-4	29.44	0.000	0.000	0.000	0.000	0.000	0.000	0.000	0.000	0.000	0.000	0.082	0.013
α-Naphthol	90-15-3		0.006	0.001	0.000	0.000	0.006	0.001	0.000	0.000	0.000	0.000	0.109	0.062
β-Naphthol	135-19-3	30.47	0.001	0.000	0.000	0.000	0.003	0.001	0.000	0.000	0.001	0.000	0.244	0.048
4-Methoxyphenol	150-76-5	30.55	0.001	0.000	0.001	0.000	0.001	0.000	0.001	0.000	0.015	0.004	0.090	0.016
1,4-Dimethoxybenzene	150-78-7	31.96	0.128	0.110	0.048	0.007	0.096	0.027	0.044	0.002	0.000	0.000	0.000	0.000
Phenol	108-95-2	32.50	0.041	0.008	0.080	0.020	0.127	0.045	1.754	0.078	0.158	0.037	1.294	0.342
m-Cresol	108-39-4	33.23	0.270	0.086	0.010	0.003	0.022	0.004	0.355	0.080	0.028	0.005	0.194	0.032
p-Cresol	106-44-5	33.30	0.000	0.000	0.000	0.000	0.001	0.000	0.001	0.000	0.007	0.002	0.094	0.028
2,6-Di-t-butyl-4-methylphenol	128-37-0	33.48	0.029	0.003	0.026	0.006	0.039	0.012	0.075	0.006	0.030	0.017	0.191	0.027
4-Ethyl phenol	123-07-9	34.18	0.000	0.000	0.000	0.000	0.000	0.000	0.001	0.000	0.001	0.000	0.000	0.000
Thymol	89-83-8	34.32	0.041	0.009	0.044	0.006	0.113	0.018	2.738	0.117	0.409	0.087	2.118	0.417
2-Acetyl-4-Methylphenol	1450-72-2	34.43	0.213	0.067	0.341	0.039	0.400	0.140	0.000	0.000	0.000	0.000	0.000	0.000
2-Methoxy-4-vinylphenol	7786-61-0	34.40	0.000	0.000	0.000	0.000	0.000	0.000	3.695	0.136	0.376	0.087	2.745	0.488
Carvacrol	499-75-2	34.64	0.071	0.025	0.093	0.016	0.235	0.059	0.294	0.021	0.205	0.030	0.091	0.011
2,4-Di-tert-butylphenol	96-76-4	35.98	0.014	0.001	0.019	0.006	0.040	0.016	0.053	0.002	0.044	0.002	0.091	0.020
3,4,5-Trimethylphenol	527-54-8	36.73	0.000	0.000	0.000	0.000	0.001	0.000	0.053	0.013	0.001	0.000	0.011	0.017
2-ethyl-5-propil-phenol	72386-20-0	38.50	0.000	0.000	0.000	0.000	0.000	0.000	0.000	0.000	0.000	0.000	0.055	0.019
Carboxylic acids														
2,2-Dimethyl-propanoic acid	15784-26-6	5.09	0.026	0.008	0.063	0.015	0.027	0.009	0.039	0.004	0.080	0.031	0.001	0.000
Acetic acid	64-19-7	22.18	11.542	3.977	17.676	3.746	32.282	5.106	41.401	0.843	25.426	0.811	81.891	4.069

Formic acid	64-18-6	23.46	0.000	0.000	1.229	0.413	0.081	0.027	5.083	0.375	1.426	0.307	20.018	3.744
Propanoic acid	79-09-4	24.25	1.527	0.316	2.223	0.448	2.376	0.296	3.640	0.466	3.417	0.360	4.329	0.210
2-Methylpropanoic acid	79-31-2	24.94	0.184	0.108	0.581	0.132	1.160	0.038	3.262	0.219	1.199	0.034	6.350	0.552
Pivalic acid	75-98-9	25.18	3.168	0.976	5.076	0.971	4.794	0.414	3.751	0.348	6.739	0.485	4.682	0.463
Butanoic acid	107-92-6	26.17	1.128	0.281	1.922	0.374	5.009	0.504	21.285	1.371	3.034	0.013	8.474	0.785
Propenoic acid	79-10-7	26.31	0.001	0.000	0.001	0.000	0.001	0.000	0.072	0.014	0.025	0.005	0.456	0.093
3-Methyl-butanoic acid	503-74-2	27.03	0.554	0.378	2.380	0.328	3.722	0.789	6.664	0.127	2.386	0.381	14.230	1.408
2-Methyl-butanoic acid	116-53-0	27.04	0.532	0.363	1.382	0.210	1.948	0.413	3.912	0.137	1.685	0.240	12.448	1.320
Pentanoic acid	109-52-4	28.33	0.329	0.139	0.532	0.140	0.833	0.028	1.382	0.320	1.176	0.132	2.075	0.328
2-Methyl-pentanoic acid	97-61-0	28.92	0.006	0.003	0.043	0.003	0.019	0.005	0.016	0.005	0.030	0.002	0.212	0.032
Crotonic acid	3724-65-0	28.94	0.003	0.001	0.029	0.003	0.015	0.004	0.033	0.002	0.021	0.003	0.117	0.014
2-Methyl-2-butenoic acid	13201-46-2	29.25	0.018	0.001	0.005	0.001	0.032	0.008	0.008	0.012	0.000	0.000	0.096	0.014
3-Methyl-pentanoic acid	105-43-1	29.42	0.029	0.018	0.039	0.007	0.306	0.135	0.365	0.043	0.338	0.087	0.431	0.060
3-Methyl-2-butenoic acid	541-47-9	29.36	0.034	0.018	0.060	0.006	0.112	0.049	0.439	0.048	0.330	0.024	0.812	0.150
4-Methyl-pentanoic acid	646-07-1	29.54	0.000	0.000	0.000	0.000	0.040	0.024	0.080	0.014	0.056	0.013	0.154	0.021
Hexanoic acid	142-62-1	30.33	0.261	0.122	1.026	0.320	1.547	0.823	18.796	2.034	1.675	0.333	7.978	1.125
2-Methyl-(E)-2-pentenoic acid	16957-70-3	30.82	0.000	0.000	0.000	0.000	0.000	0.000	0.001	0.000	0.000	0.000	0.070	0.025
Heptanoic acid	111-14-8	31.96	0.115	0.138	0.116	0.016	0.163	0.046	1.454	0.086	0.272	0.039	2.772	0.434
3-Hexenoic acid	1577-18-0	31.99	0.000	0.000	0.004	0.001	0.010	0.003	0.756	0.100	0.009	0.002	0.494	0.074
2-Hexenoic acid	13419-69-7	32.02	0.227	0.219	0.139	0.019	0.000	0.000	0.033	0.007	0.000	0.000	0.083	0.014
Octanoic acid	124-07-2	33.05	0.006	0.000	0.023	0.014	0.027	0.030	7.906	2.545	0.045	0.007	3.912	0.524
7-Octenoic acid	18719-24-9	33.62	0.000	0.000	0.000	0.000	0.000	0.000	0.062	0.017	0.001	0.000	0.238	0.035
Hexa-2,4-Dienoic acid	110-44-1	33.77	0.000	0.000	0.000	0.000	0.000	0.000	0.303	0.046	0.000	0.000	0.000	0.000
Hexa-2,4-Dienoic acid isomer		33.82	0.000	0.000	0.000	0.000	0.000	0.000	13.234	1.586	0.536	0.216	0.001	0.000
Nonanoic acid	112-05-0	34.11	0.001	0.000	0.000	0.000	0.059	0.041	1.998	0.984	0.107	0.028	6.413	1.356
n-Decanoic acid	334-48-5	35.34	0.000	0.000	0.000	0.000	0.000	0.000	0.997	0.868	0.011	0.005	0.155	0.085
2-Nonenoic acid	3760-11-0	35.57	0.000	0.000	0.000	0.000	0.000	0.000	0.000	0.000	0.016	0.003	0.198	0.086
Nerolic acid	4613-38-1	36.30	0.000	0.000	0.000	0.000	0.000	0.000	0.054	0.010	0.000	0.000	0.449	0.101
Benzoic acid	65-85-0	38.26	0.029	0.010	0.218	0.038	0.088	0.028	10.260	0.676	0.183	0.076	10.993	1.652
Dodecanoic acid	143-07-7	39.11	0.000	0.000	0.000	0.000	0.000	0.000	0.108	0.103	0.000	0.000	0.000	0.000
Phenylacetic acid	103-82-2	40.85	0.000	0.000	0.000	0.000	0.000	0.000	0.032	0.005	0.001	0.000	0.177	0.060
Esters														
Methyl acetate	79-20-9	2.37	0.000	0.000	0.000	0.000	0.000	0.000	0.070	0.015	0.077	0.021	0.107	0.051
Ethyl acetate	141-78-6	3.01	0.238	0.024	0.296	0.015	0.363	0.047	0.355	0.026	0.315	0.034	0.494	0.072
Methyl hexanoate	106-70-7	14.34	0.003	0.001	0.000	0.000	0.000	0.000	0.144	0.021	0.012	0.014	0.038	0.001
Ethyl hexanoate	123-66-0	16.29	0.000	0.000	0.000	0.000	0.001	0.000	0.049	0.006	0.000	0.000	0.001	0.000
Ethyl lactate	97-64-3	19.64	0.022	0.012	0.019	0.003	0.604	0.067	0.107	0.016	0.003	0.000	0.048	0.007
4,6-Dimethyltetrahydro-2H-pyran-2-one	3720-20-5	21.92	0.024	0.010	0.036	0.012	0.041	0.008	0.052	0.015	0.022	0.003	0.052	0.009
Methyl nonanoate	1731-84-6	23.44	0.015	0.007	0.048	0.011	0.007	0.001	0.128	0.036	0.042	0.007	0.046	0.080
γ-Valerolactone	108-29-2	25.60	0.085	0.030	0.147	0.007	0.342	0.040	0.882	0.202	0.222	0.016	1.013	0.210
Methyl benzoate	93-58-3	26.06	0.001	0.000	0.001	0.000	0.000	0.000	0.014	0.004	0.001	0.000	0.083	0.024
Ethyl decanoate	110-38-3	26.59	0.000	0.000	0.000	0.000	0.000	0.000	0.250	0.171	0.000	0.000	0.000	0.000
γ-Vinyl-γ-valerolactone	1073-11-6	26.85	0.374	0.091	0.603	0.022	0.400	0.075	1.873	0.107	0.183	0.018	2.411	0.316
Ethyl benzoate	93-89-0	26.98	0.000	0.001	0.049	0.012	0.079	0.017	0.001	0.000	0.001	0.000	0.001	0.000
γ-Caprolactone	695-06-7	27.47	0.039	0.065	0.250	0.024	0.372	0.066	0.796	0.054	0.196	0.171	1.097	0.185
γ-Crotonlactone	497-23-4	28.45	0.001	0.000	0.001	0.000	0.023	0.001	0.023	0.006	0.033	0.008	0.056	0.008
Methyl salicylate	119-36-8	29.01	0.071	0.022	0.020	0.003	0.047	0.011	0.624	0.284	0.056	0.048	0.052	0.005
Laurate	111-82-0	29.73	0.001	0.000	0.001	0.000	0.000	0.000	0.452	0.229	0.003	0.001	0.001	0.000

Phenethyl acetate	103-45-7	29.83	0.001	0.000	0.000	0.000	0.029	0.006	0.020	0.006	0.001	0.000	0.000	0.000
Pantolactone	52126-90-6	32.71	0.001	0.000	0.001	0.000	0.042	0.016	1.189	0.244	0.191	0.129	0.414	0.158
1-(4-Isopropylphenyl)-2-methylpropyl acetate	ID#: 311526	33.09	0.070	0.004	0.069	0.008	0.166	0.020	0.001	0.000	0.034	0.005	0.001	0.000
Dihydropentalactone	21950-33-4	33.54	0.019	0.005	0.021	0.004	0.000	0.000	0.000	0.000	0.000	0.000	0.000	0.000
Methyl citronellate	2270-60-2	35.04	0.000	0.000	0.000	0.000	0.000	0.000	0.000	0.000	0.000	0.000	0.174	0.043
Diethyl phthalate	84-66-2	36.81	0.021	0.003	0.022	0.001	0.001	0.000	0.032	0.009	0.029	0.022	0.127	0.006
Sparassol	520-43-4	37.01	0.018	0.010	0.010	0.002	0.013	0.004	0.023	0.004	0.012	0.005	0.011	0.001
Butyl hexadecanoate	111-06-8	38.18	0.000	0.000	0.000	0.000	0.000	0.000	0.001	0.000	0.000	0.000	0.316	0.311
Diisobutyl phthalate	84-69-5	40.41	0.009	0.001	0.001	0.000	0.001	0.000	0.022	0.010	0.014	0.007	0.081	0.027
Benzyl benzoate	120-51-4	42.59	0.000	0.000	0.001	0.000	0.000	0.000	0.032	0.010	0.000	0.000	0.031	0.009
Stearate	123-95-5	43.93	0.000	0.000	0.000	0.000	0.000	0.000	0.055	0.003	0.022	0.002	0.242	0.164
Eters														
Diethyl ether	60-29-7	2.40	0.000	0.000	0.000	0.000	0.027	0.002	0.000	0.000	0.000	0.000	0.000	0.000
Dill ether	70786-44-6	23.71	0.114	0.030	0.136	0.016	0.135	0.017	0.624	0.111	0.074	0.015	0.409	0.074
Furanoids														
2-Methylfuran	534-22-5	2.81	0.000	0.000	0.000	0.000	0.000	0.000	0.000	0.000	0.000	0.000	0.364	0.032
3-Methylfuran	930-27-8	3.12	0.184	0.057	0.145	0.008	0.285	0.009	0.426	0.022	0.256	0.021	1.474	0.043
2-Ethylfuran	3208-16-0	4.21	0.001	0.000	0.001	0.000	0.001	0.000	0.001	0.000	0.001	0.000	0.166	0.030
z-Rose-oxide	16409-43-1	19.82	0.011	0.005	0.027	0.008	0.003	0.001	0.112	0.026	0.072	0.009	0.306	0.032
Menthofuran	494-90-6	23.07	0.001	0.000	0.001	0.000	0.000	0.000	0.000	0.000	0.000	0.000	3.652	0.418
4,7-Dimethyl-benzofuran	28715-26-6	27.68	0.001	0.000	0.195	0.067	0.000	0.000	0.001	0.000	0.000	0.000	0.747	0.155
4,5-Dimethyl-2-formylfuran	52480-43-0	32.16	0.056	0.013	3.534	1.189	0.340	0.104	3.059	0.390	6.247	1.765	0.838	0.227
2,3-Dihydro-benzofuran	496-16-2	37.09	0.001	0.000	0.029	0.002	0.028	0.014	0.506	0.059	0.015	0.005	0.709	0.113
Hydrocarbons														
n-Octane	111-65-9	2.19	0.122	0.037	0.254	0.055	0.064	0.011	0.668	0.161	0.210	0.033	2.146	0.229
1-Octene	111-66-0	2.52	0.001	0.000	0.064	0.006	0.039	0.005	0.184	0.007	0.051	0.004	0.366	0.078
2,2,3-Trimethyl-hexane	16747-25-4	7.70	0.051	0.008	0.088	0.024	0.816	0.155	0.742	0.804	2.304	0.278	1.853	0.387
2,3,4-Trimethyl-decane	62238-15-7	8.18	0.000	0.000	0.043	0.012	0.068	0.003	1.320	1.067	0.084	0.015	0.236	0.011
2,8-dimethyl-undecane	17301-25-6	8.21	0.031	0.006	0.000	0.000	0.000	0.000	0.000	0.000	0.000	0.000	0.000	0.000
n-Undecane	1120-21-4	10.03	0.001	0.000	0.016	0.007	0.011	0.001	0.057	0.033	0.021	0.001	0.040	0.003
1,3-Cyclopentadiene	542-92-7	14.65	0.256	0.072	0.000	0.000	0.000	0.000	0.001	0.000	0.000	0.000	0.175	0.028
E- 2-Methyl-3-penten-1-ol	629-50-5	18.67	0.012	0.004	0.019	0.006	0.015	0.001	0.038	0.012	0.016	0.006	0.054	0.013
Tetradecane	629-59-4	21.42	0.000	0.000	0.000	0.000	0.076	0.010	0.068	0.029	0.022	0.011	0.098	0.030
1,4-Dimethyl-4-acetyl-1-cyclohexene	43219-68-7	23.85	0.604	0.135	0.605	0.060	1.028	0.114	0.713	0.020	0.353	0.033	0.166	0.012
Dodecane	112-40-3	15.15	0.041	0.017	0.125	0.025	0.001	0.000	0.343	0.042	0.055	0.012	0.185	0.039
Ketones														
Acetone	67-64-1	2.27	0.463	0.012	0.508	0.880	2.204	0.103	1.935	0.130	3.097	0.142	0.000	0.000
3-Methyl-2-butanone	563-80-4	3.61	0.000	0.000	0.000	0.000	0.000	0.000	0.000	0.000	0.000	0.000	0.036	0.012
2-Pentanone	107-87-9	4.68	0.000	0.000	0.000	0.000	0.000	0.000	0.023	0.006	0.001	0.000	0.048	0.031
Diacetyl	431-03-8	4.86	0.119	0.026	0.248	0.025	0.387	0.047	0.862	0.043	0.484	0.016	14.201	0.426
E-3-Penten-2-one	3102-33-8	11.09	0.001	0.000	0.000	0.000	0.000	0.000	0.007	0.011	0.057	0.027	0.139	0.012
4-Methyl-3-penten-2-one	141-79-7	11.39	0.001	0.000	0.000	0.000	0.001	0.000	0.014	0.001	0.014	0.007	0.067	0.004
2-Heptanone	110-43-0	13.93	0.024	0.009	0.011	0.003	0.008	0.001	0.063	0.008	0.027	0.004	0.072	0.001
6-Methyl-2-heptanone	928-68-7	16.24	0.050	0.025	0.064	0.007	0.013	0.000	0.002	0.001	0.021	0.004	0.048	0.011
2-Methyltetrahydro-3-furanone	3188-00-9	17.02	0.001	0.000	0.001	0.000	0.001	0.000	0.024	0.007	0.001	0.000	0.061	0.011
3-Hydroxy-2-butanone	513-86-0	17.64	0.154	0.069	0.227	0.023	0.395	0.030	0.599	0.063	0.209	0.123	2.462	0.093
2-Octanone	111-13-7	17.82	0.010	0.005	0.007	0.002	0.000	0.000	0.024	0.005	0.011	0.003	0.054	0.012

1-Hydroxyacetone	116-09-6	18.07	0.097	0.054	0.234	0.055	0.304	0.040	0.910	0.118	0.709	0.196	2.389	0.236
2,6-Dimethyl-4-hepten-3-one	56259-14-4	18.90	0.000	0.000	0.001	0.000	0.000	0.000	0.000	0.000	0.005	0.001	0.027	0.003
6-Methyl-5-hepten-2-one	110-93-0	19.42	0.010	0.002	0.012	0.003	0.008	0.001	0.024	0.004	0.011	0.001	0.038	0.005
4-Hydroxy-4-methyl-2-pentanon	123-42-2	19.95	0.000	0.000	0.006	0.002	0.010	0.004	0.004	0.001	0.004	0.000	0.001	0.000
Tetrahydro-3,5-dimethyl-2h-pyran-2-one	3290-57-1	20.27	0.065	0.023	0.008	0.001	0.094	0.019	0.014	0.001	0.008	0.004	0.008	0.001
2-Nonanone	821-55-6	20.84	0.048	0.014	0.000	0.000	0.000	0.000	0.262	0.048	0.006	0.000	0.088	0.039
3,5,5-Trimethyl-3-Cyclohexen-1-one	471-01-2	21.19	0.000	0.000	0.018	0.011	0.001	0.000	0.129	0.011	0.032	0.004	0.058	0.007
2-Cyclohexen-1-one	930-68-7	21.67	0.000	0.000	0.062	0.007	0.073	0.010	0.001	0.000	0.000	0.000	0.457	0.074
3-Methyl-3-cyclohexen-1-one	31883-98-4	21.63	0.001	0.000	0.020	0.001	0.242	0.033	0.165	0.007	0.143	0.008	0.063	0.012
2-Acetylfuran	1192-62-7	23.50	0.031	0.009	0.316	0.055	0.655	0.141	0.933	0.071	0.000	0.000	2.440	0.677
3-Methyl-2-cyclopenten-1-one	2758-18-1	23.59	0.159	0.046	0.077	0.018	0.152	0.048	0.001	0.000	0.404	0.044	0.000	0.000
α-Isophorone	78-59-1	25.29	0.001	0.000	0.001	0.000	0.001	0.000	2.643	0.246	0.001	0.000	0.913	0.194
Acetophenone	98-86-2	26.55	0.001	0.000	0.001	0.000	0.001	0.000	0.001	0.000	0.001	0.000	1.829	0.130
3,4-Dimethylacetophenone	3637 01 2	27.18	0.001	0.000	0.001	0.000	0.023	0.005	0.000	0.000	0.001	0.000	0.446	0.080
4-Oxoisophorone	1125-21-9	27.38	0.157	0.229	0.982	0.123	0.815	0.145	2.104	0.144	0.413	0.359	2.620	0.313
Verbenone	80-57-9	27.60	0.931	0.677	0.093	0.004	0.057	0.008	0.022	0.002	0.009	0.001	0.057	0.010
3,4-Dimethyl-2,5-Furandione	766-39-2	27.98	0.000	0.000	0.000	0.000	0.000	0.000	0.000	0.000	0.000	0.000	0.456	0.065
3,3,5-Trimethyl-Cyclohexanone	873-94-9	28.56	0.001	0.000	0.074	0.003	0.079	0.008	0.001	0.000	0.001	0.000	0.079	0.007
cyclopentane-1,2-dione	3008-40-0	28.81	0.000	0.000	0.001	0.000	0.001	0.000	0.013	0.006	0.027	0.010	0.084	0.024
3-Methylacetophenone	585-74-0	28.96	0.014	0.001	0.058	0.006	0.028	0.008	0.250	0.206	0.026	0.001	0.643	0.036
2,2,6-Trimethyl-1,4-cyclohexanedione	20547-99-3	29.00	0.005	0.002	0.012	0.003	0.033	0.008	0.396	0.060	0.026	0.022	0.133	0.032
Tetrahydro-6-methyl-2h-pyran-2-one	823-22-3	29.17	0.001	0.000	0.038	0.006	0.117	0.028	0.016	0.001	0.001	0.000	0.646	0.096
β-Damascenone	23696-85-7	29.93	0.106	0.026	0.558	0.083	0.108	0.018	2.487	0.238	0.482	0.095	0.644	0.118
(+)-Isopiperitenone	16750-82-6	30.13	0.047	0.010	0.036	0.004	0.019	0.005	0.037	0.014	0.019	0.002	0.060	0.008
5-Methyl-5H-furan-2-one	591-11-7	30.93	0.000	0.000	0.000	0.000	0.040	0.012	0.093	0.024	0.065	0.023	0.001	0.000
2-Hydroxy-3,5,5-trimethyl-2-cyclohexen-1,4-dione	35692-98-9	32.76	0.001	0.000	0.001	0.000	0.019	0.009	0.032	0.002	0.010	0.003	0.000	0.000
1-Carvone	99-49-0	34.62	0.000	0.000	0.000	0.000	0.000	0.000	0.000	0.000	0.000	0.000	1.441	0.192
Hydroxydihydromaltol	28564-83-2	35.25	0.000	0.000	0.000	0.000	0.000	0.000	0.000	0.000	0.000	0.000	0.307	0.095
8-Hydroxycarvotanacetone	7712-46-1	36.15	0.001	0.000	0.000	0.000	0.038	0.016	0.092	0.012	0.052	0.034	0.127	0.056
4′-Isopropylacetophenone	645-13-6	38.66	0.000	0.000	0.000	0.000	0.001	0.000	0.247	0.051	0.024	0.007	0.158	0.126
Nitrogenates														
N-Methylpropylamine	627-35-0	4.35	0.000	0.000	0.001	0.000	0.000	0.000	0.000	0.000	0.038	0.006	0.000	0.000
2,5-Dimethyl-pyrazine	123-32-0	18.79	0.000	0.000	0.002	0.004	0.000	0.000	0.564	0.057	0.024	0.003	0.037	0.008
2-Methyl-propanamide	563-83-7	29.39	0.000	0.000	0.007	0.003	0.000	0.000	0.366	0.095	0.001	0.000	0.021	0.006
Butyramide	541-35-5	31.06	0.000	0.000	0.000	0.000	0.000	0.000	0.051	0.010	0.000	0.000	0.001	0.000
Isovaleramide	541-46-8	31.39	0.000	0.000	0.000	0.000	0.001	0.000	0.561	0.078	0.038	0.007	0.000	0.000
Phenylacetonitrile	140-29-4	31.58	0.030	0.017	0.122	0.017	0.114	0.032	0.049	0.001	0.051	0.011	3.112	0.345
2-Pyrrolidone	616-45-5	32.71	0.001	0.000	0.000	0.000	0.000	0.000	0.001	0.000	0.000	0.000	0.086	0.047
2'-Aminoacetophenone	551-93-9	34.65	0.000	0.000	0.001	0.000	0.000	0.000	0.001	0.000	0.146	0.023	2.567	0.342
n-(2-Acetylphenyl)formamide	5257 06 7	40.58	0.000	0.000	0.000	0.000	0.000	0.000	0.000	0.000	0.000	0.000	0.750	0.217
Sulfurated														
Dimethyl sulfide	75-18-3	1.90	0.708	0.062	0.484	0.029	0.674	0.091	1.823	0.091	0.793	0.152	3.607	0.173
Dimethyl sulfoxide	67-68-5	24.57	0.008	0.006	0.021	0.000	0.019	0.005	0.001	0.000	0.001	0.000	0.061	0.033
Dimethylsulfone	67-71-0	31.09	0.050	0.005	0.024	0.001	0.049	0.013	0.304	0.064	0.065	0.023	0.237	0.040
Terpenes														
β-Pinene	127-91-3	9.61	0.000	0.000	0.000	0.000	0.040	0.001	0.079	0.023	0.068	0.013	0.084	0.027
Sabinene	3387-41-5	10.49	0.001	0.000	0.000	0.000	0.080	0.013	0.207	0.004	0.189	0.015	0.183	0.024

α-Phellandrene	99-83-2	12.77	0.001	0.000	0.001	0.000	0.001	0.000	0.001	0.000	0.001	0.000	0.033	0.008
β-Myrcene	123-35-3	13.24	0.008	0.002	0.003	0.005	0.001	0.000	0.039	0.008	0.040	0.013	0.040	0.004
α-Terpinene	99-86-5	13.49	0.031	0.013	0.009	0.006	0.016	0.003	0.061	0.014	0.028	0.007	0.260	0.023
Limonene	138-86-3	14.37	0.190	0.053	0.333	0.122	0.051	0.009	0.232	0.004	0.193	0.214	0.135	0.027
1, 8-Cineol	470-82-6	14.72	0.172	0.063	0.105	0.024	0.045	0.007	0.161	0.019	0.081	0.006	0.203	0.027
Herboxide	13679-86-2	15.18	0.057	0.023	0.000	0.000	0.128	0.016	0.000	0.000	0.064	0.009	0.000	0.000
trans-3(10)-caren-2-ol	6909-15-5	15.35	0.046	0.014	0.002	0.003	0.049	0.009	0.493	0.045	0.152	0.016	5.417	0.118
γ-Terpinene	99-85-4	16.37	0.026	0.012	0.005	0.003	0.077	0.011	0.284	0.037	0.087	0.009	0.687	0.081
Herboxide isomer		16.42	0.171	0.077	0.086	0.003	0.163	0.023	0.474	0.069	0.091	0.009	0.000	0.000
E-β-Ocimene	3779-61-1	16.85	0.001	0.000	0.001	0.000	0.001	0.000	0.034	0.005	0.001	0.000	0.001	0.000
m-Cymene	535-77-3	17.22	0.190	0.095	0.181	0.016	0.186	0.028	0.423	0.037	0.166	0.028	0.799	0.100
Terpinolene	586-62-9	17.67	0.001	0.000	0.001	0.000	0.137	0.016	0.478	0.033	0.186	0.109	0.087	0.007
p-Mentha-1,3,8-triene	21195-59-5	20.86	0.001	0.001	0.001	0.000	0.056	0.028	0.000	0.000	0.028	0.009	0.067	0.018
Cymenene	1195-32-0	21.97	0.053	0.025	0.069	0.008	0.183	0.034	0.273	0.019	0.162	0.030	11.533	0.797
Carvyl acetate	97-42-7	22.04	0.132	0.046	0.153	0.014	0.281	0.042	0.175	0.010	0.076	0.034	0.127	0.020
cis-Linalool oxide	5989-33-3	22.22	1.398	0.286	1.330	0.162	1.372	0.131	4.031	0.124	0.000	0.000	1.731	0.161
trans-Linalool oxide	34995-77-2	22.85	1.221	0.394	1.467	0.141	1.770	0.222	3.589	0.028	1.072	0.042	1.769	0.127
Dihydromyrcenol	53219-21-9	22.96	0.008	0.000	0.000	0.000	0.019	0.005	0.077	0.025	0.014	0.001	0.001	0.000
Linalool	78-70-6	24.68	2.180	0.405	4.134	0.302	1.061	0.078	19.977	1.606	4.835	0.351	11.140	0.707
(-)-*trans*-Pinocarvone	19890-00-7	24.85	4.689	0.662	4.161	0.291	1.505	0.231	4.274	0.354	2.526	0.211	2.339	0.290
4-Terpineol	562-74-3	25.71	1.045	0.192	0.887	0.099	0.335	0.046	2.280	0.417	0.348	0.037	2.785	0.283
1,8-Menthadien-4-ol	3419-02-1	27.44	0.719	0.021	1.127	0.117	1.652	0.293	1.161	0.092	0.501	0.436	0.451	0.110
α-Terpineol	10482-56-1	27.62	0.371	0.353	0.764	0.059	0.653	0.093	3.802	0.324	1.057	0.061	2.221	0.239
Valencene	4630 03 7	28.02	0.000	0.000	0.000	0.000	0.000	0.000	0.523	0.199	0.039	0.006	0.000	0.000
p-Mentha-1,5-dien-8-ol	1686-20-0	28.17	0.214	0.026	0.155	0.031	0.238	0.087	0.723	0.062	0.226	0.039	0.235	0.046
(+)-δ-Cadinene	483-76-1	28.86	0.043	0.005	0.052	0.002	0.022	0.012	0.197	0.056	0.020	0.008	0.048	0.005
p-Mentha-1,5-dien-8-ol isomer	1686-20-0	29.20	0.186	0.031	0.213	0.038	0.604	0.146	0.083	0.023	0.021	0.008	0.465	0.115
trans-3-caren-2-ol	6909-16-6	29.68	0.103	0.017	0.141	0.031	0.174	0.026	0.104	0.020	0.086	0.012	0.202	0.033
Calamenene	483-77-2		0.001	0.000	0.000	0.000	0.001	0.000	0.032	0.018	0.001	0.000	0.026	0.002
p-Cymen-7-ol	536-60-7	30.42	0.136	0.032	0.206	0.030	1.861	0.446	0.868	0.034	1.295	0.180	3.665	0.582
α-Calacorene	21391-99-1	31.56	0.071	0.058	0.050	0.007	0.039	0.011	0.085	0.012	0.030	0.008	0.035	0.006
Eugenol	97-53-0	34.12	0.000	0.000	0.053	0.006	0.000	0.000	0.210	0.049	0.028	0.011	0.001	0.000
10-epi-γ-eudesmol	1209-71-8	34.20	0.000	0.000	0.001	0.000	0.104	0.035	0.001	0.000	0.070	0.024	0.000	0.000
Guaiazulene	489-84-9	34.82	0.001	0.000	0.028	0.004	0.074	0.025	0.001	0.000	0.060	0.014	0.001	0.000
(+)-β-Eudesmol	473-15-4	34.92	0.000	0.000	0.000	0.000	0.064	0.030	0.001	0.000	0.111	0.056	0.001	0.000
cis-Isoeugenol	5932-68-3	36.42	0.000	0.000	0.000	0.000	0.000	0.000	0.053	0.008	0.001	0.000	0.001	0.000
Other														
1-Chloro-octane	111-85-3	16.65	0.033	0.017	0.020	0.002	0.013	0.003	0.022	0.005	0.021	0.012	0.000	0.000
Cyclooctene oxide	286-62-4	21.78	0.001	0.000	0.053	0.006	0.053	0.007	0.001	0.000	0.001	0.000	0.323	0.031

Table S4. Results regarding metabolomic identification of phenolic compounds (Rt, retention time).

RT [min]	Name	Formula	exact mass
	**Flavanone**		
**17,6**	4'-Hydroxyflavanone	$C_{15}H_{12}O_3$	274,1777
**19,3**	Flavanone	$C_{15}H_{12}O_2$	225,0910
**19,5**	Naringenin	$C_{15}H_{12}O_5$	272,0682
**23,6**	Isosakuranetin	$C_{16}H_{14}O_5$	287,0914
**28,7**	Sakuranetin	$C_{16}H_{14}O_5$	287,0914
	**Flavone**		
**20,2**	Hispidulin	$C_{16}H_{12}O_6$	300,0630
**20,4**	6,7-Dihydroxyflavone	$C_{15}H_{10}O_4$	254,0579
**20,7**	5,7,8-Trihydroxyflavone	$C_{15}H_{10}O_5$	270,0522
**21,3**	Luteolin	$C_{15}H_{10}O_6$	286,0477
**21,9**	5-Hydroxy-4',6,7-trimethoxyflavone	$C_{18}H_{16}O_6$	328,0943
**22,4**	Hispidulin	$C_{16}H_{12}O_6$	300,0630
**22,4**	7,8-Dihydroxyflavone	$C_{15}H_{10}O_4$	254,0579
**22,6**	3',4'-Dihydroxyflavone	$C_{15}H_{10}O_4$	254,0579
**22,6**	Tricin	$C_{17}H_{14}O_7$	330,0736
**23,5**	Pectolinarigenin	$C_{17}H_{14}O_6$	314,0787
**26,2**	5,6,7-Trihydroxyflavone	$C_{15}H_{10}O_5$	270,0522
**26,5**	Tetramethylscutellarein	$C_{19}H_{18}O_6$	342,1099
	**Flavonol**		
**11,0**	5-Deoxykaempferol	$C_{15}H_{10}O_5$	270,0520
**18,3**	Fisetin	$C_{15}H_{10}O_6$	286,0470
**19,3**	Galangin	$C_{15}H_{10}O_5$	270,0520
**19,7**	Quercetin	$C_{15}H_{10}O_7$	302,0422
**20,5**	Kaempferol	$C_{15}H_{10}O_6$	286,0473
**20,6**	3-Methylquercetin	$C_{16}H_{12}O_7$	316,0580
**24,9**	Robinetin	$C_{15}H_{10}O_7$	302,0421
**25,1**	Kaempferol 3,7,4'-trimethyl ether	$C_{18}H_{16}O_6$	328,0943
	**Isoflavone**		
**17,3**	Daidzein	$C_{15}H_{10}O_4$	254,0579
**18,8**	Vestitone	$C_{16}H_{14}O_5$	286,0838
**19,3**	3'-Hydroxybiochanin A	$C_{16}H_{12}O_6$	300,0634
**20,0**	Biochanin A	$C_{16}H_{12}O_5$	284,0685
**21,0**	2'-Hydroxyformononetin	$C_{16}H_{12}O_5$	284,0685
**22,3**	Formononetin	$C_{16}H_{12}O_4$	268,0735
**23,0**	3'-Hydroxyprunetin	$C_{16}H_{12}O_6$	300,0634
**23,5**	Pisatin	$C_{17}H_{14}O_6$	314,0787
**24,2**	Glycitein	$C_{16}H_{12}O_5$	284,0681
**24,3**	Prunetin	$C_{16}H_{12}O_5$	284,0685
**25,0**	Genistein	$C_{17}H_{10}O_5$	270,0526
**25,3**	4',7-Dimethoxyisoflavone	$C_{17}H_{14}O_4$	282,0892

**26,4**	Luteone	$C_{20}H_{18}O_6$	354,1099
	**Phenolic acid and derivates**		
**3,7**	protocatechuic acid	$C_7H_6O_4$	154,0263
**10,2**	Dihydro-p-coumaric acid	$C_9H_{10}O_3$	166,0627
**11,0**	dihydroconiferyl alcohol glucoside	$C_{16}H_{24}O_8$	344,1468
**12,9**	dihydroconiferyl alcohol glucoside	$C_{16}H_{24}O_8$	344,1466
**12,7**	3,4,5-Trimethoxycinnamic acid	$C_{12}H_{14}O_5$	238,0838
**13,0**	p-coumaric acid	$C_9H_8O_3$	164,0471
**13,5**	Cinnamic acid	$C_9H_8O_2$	148,0522
**13,8**	Ferulic acid	$C_{10}H_{10}O_4$	194,0575
**14,4**	4-Methoxycinnamic acid	$C_{10}H_{10}O_3$	178,0627
**15,5**	Salicylic acid	$C_7H_6O_3$	138,0314
**19,8**	tricoumaroyl spermidine	$C_{34}H_{37}N_3O_6$	583,2678
**27,8**	Coniferyl ferulate	$C_{20}H_{20}O_6$	356,1257
	**Quinoline**		
**5,9**	8-Hydroxyquinoline	C_9H_7NO	145,0525
**7,9**	2-Hydroxyquinoline	C_9H_7NO	145,0525
**9,4**	2-Quinolinecarboxylic acid	$C_{10}H_7NO_2$	173,0474
**14,1**	6-Hydroxyquinoline	C_9H_7NO	145,0525
	**Tryptophan metabolite**		
**7,9**	3-hydroxyquinaldic acid	$C_{10}H_7NO_3$	189,0423
**10,7**	5-Hydroxyindole-3-acetic acid	$C_{10}H_9NO_2$	191,0579
**13,6**	4-Aminobenzoic acid	$C_7H_7NO_2$	137,0474
**14,1**	Kynurenic acid	$C_{10}H_7NO_3$	189,0423
	**Coumarine**		
**18,8**	Oxypeucedanin	$C_{16}H_{14}O_5$	286,0838
**25,3**	4-Ethyl-7-hydroxy-3-(p-methoxyphenyl)coumarin	$C_{18}H_{16}O_4$	296,1044
	**Catechol metabolite**		
**2,4**	Guaiacol	$C_7H_8O_2$	124,0523
	**Dopamine metabolite**		
**2,4**	3,4-dihydroxyphenylacetic acid	$C_8H_8O_4$	168,0421
	**Carotenoid sesquiterpenoid**		
**13,4**	Phaseic acid	$C_{15}H_{20}O_5$	280,1306
	**Sesquiterpene**		
**11,0**	Artemisinin	$C_{15}H_{22}O_5$	282,1463
	**Glucuronated terpenoide**		
**17,4**	2-Hydroxy-2-(4-methyl-2-oxocyclohexyl)propyl hexopyranoside	$C_{16}H_{28}O_8$	348,1780
**18,8**	Dihydroxyfenchone 6 - O - D-glucoside	$C_{16}H_{26}O_8$	346,1624
	**Fatty acid and derivatives**		
**18,8**	4-Hydroxynonenal	$C_9H_{16}O_2$	156,1148
**19,6**	Dodecanedioic acid	$C_{12}H_{22}O_4$	230,1514
**22,6**	9,12,13-Trihydroxy-10,15-octadecadienoic acid	$C_{18}H_{32}O_5$	328,2246
**23,0**	Traumatic Acid	$C_{12}H_{20}O_4$	228,1358
**23,3**	3-Hydroxytetradecanedioic acid	$C_{14}H_{26}O_5$	274,1777
**23,5**	pinellic acid	$C_{18}H_{34}O_5$	330,2403
**24,0**	12-Hydroxylauric acid	$C_{12}H_{24}O_3$	216,1720

**26,2**	8-Hydroxyhexadecanedioic acid	$C_{16}H_{30}O_5$	302,2088
**26,7**	10,16-Dihydroxyhexadecanoic acid	$C_{16}H_{32}O_4$	288,2297
**28,7**	9,10-Dihydroxystearic acid	$C_{18}H_{36}O_4$	316,2609
**29,3**	Juniperic acid	$C_{16}H_{32}O_3$	272,2348
**32,3**	Elaidolinolenic acid	$C_{18}H_{30}O_2$	278,2241
**34,1**	Palmitic Acid	$C_{16}H_{32}O_2$	256,2399
**34,3**	Oleic acid	$C_{18}H_{34}O_2$	282,2555
	**linear dicarboxilic acid**		
**13,6**	Suberic acid	$C_8H_{14}O_4$	174,0888
**14,0**	3-Hydroxysebacic acid	$C_{10}H_{18}O_5$	218,1150
**19,7**	Sebacic acid	$C_{10}H_{18}O_4$	202,1203
	**Antimicrobial Peptide**		
**26,9**	Heliomycin	$C_{22}H_{16}O_6$	376,0943
	**N-acyl-aminoacids**		
**1,3**	N-Phenylacetylglutamic acid	$C_{13}H_{15}N_{O5}$	265,0947
**12,3**	N-Acetyl-L-phenylalanine	$C_{11}H_{13}N_{O3}$	207,0892

Figure S1. Most important metabolites for each honey.

Compounds dominant in F

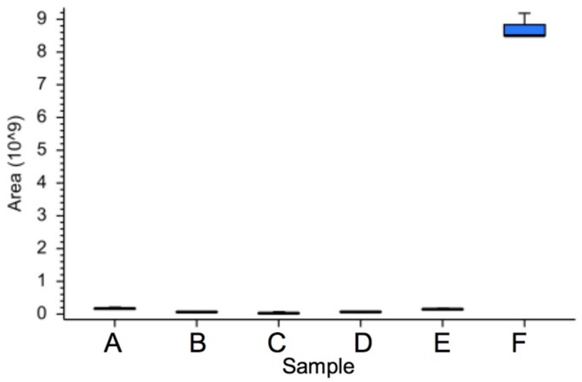

Kynurenic acid

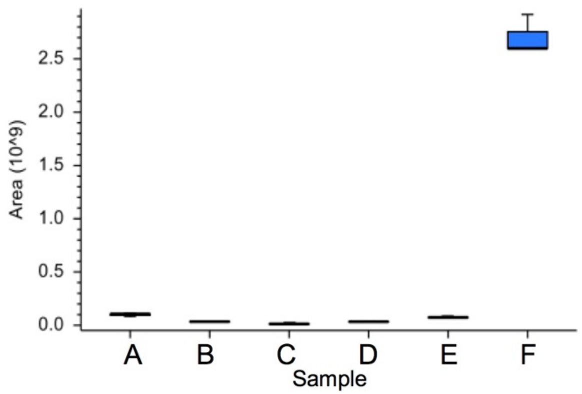

3-hydroxyquinaldic acid

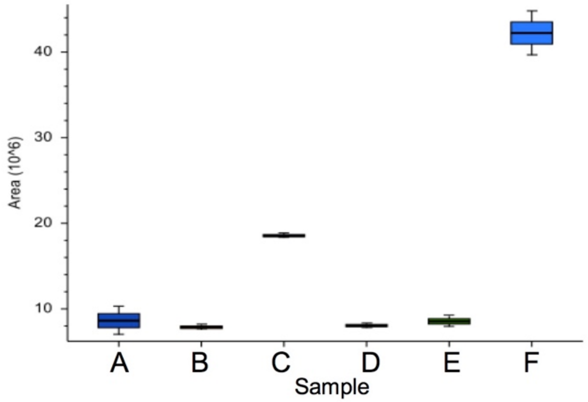

Traumatic acid

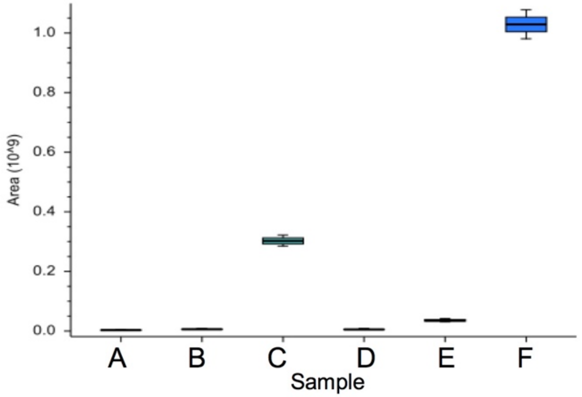

Dihydroconiferyl alcohol glucoside

Compounds dominant in C

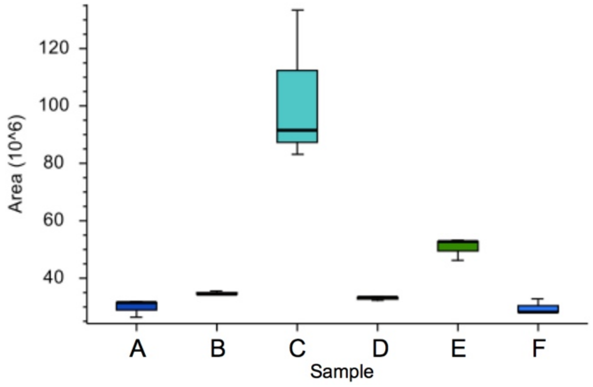

Dihydroxyfenchone 6 -O -D-glucoside (3-(Hydroxymethyl)-3-methyl-2-oxobicyclo[2.2.1]hept-1-yl]methyl hexopyranoside)

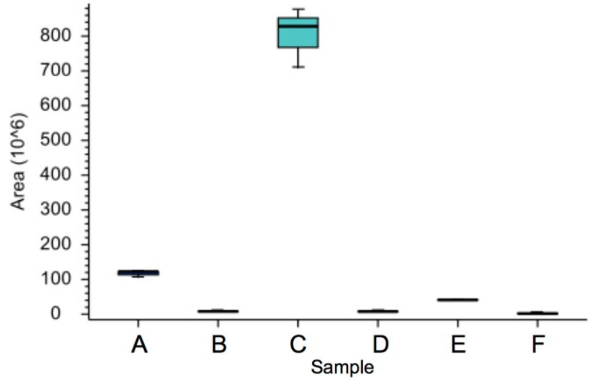

tricin

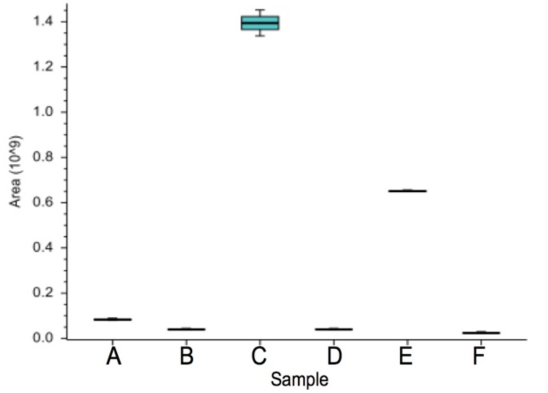

Luteolin

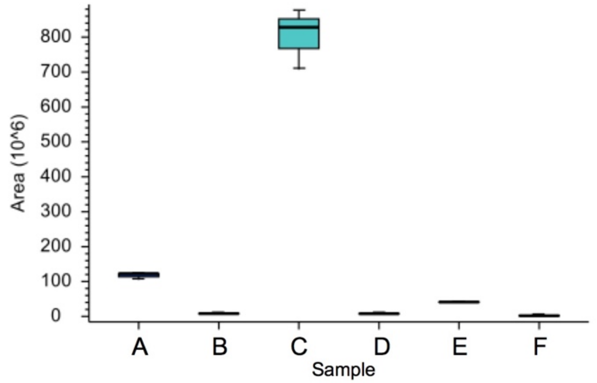

Pectolinarigenin

Compounds dominant in C and E

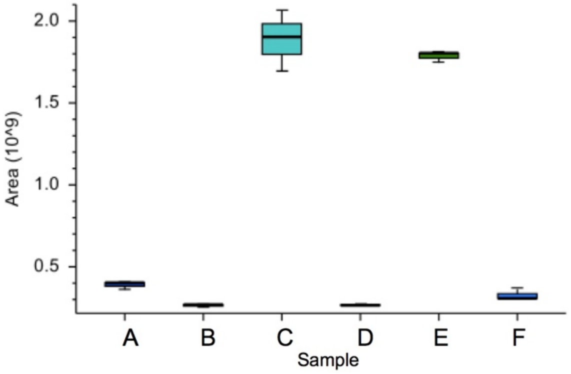

Naringenin

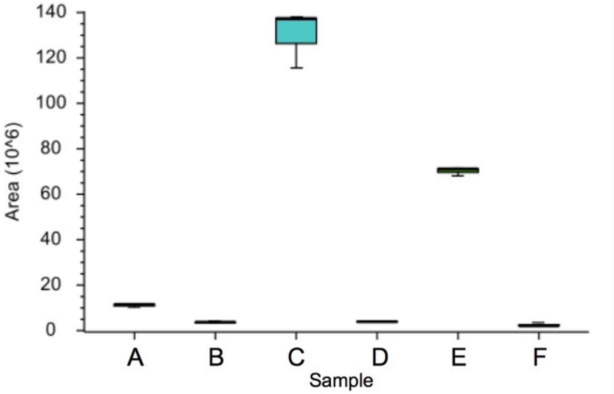

Kempherol

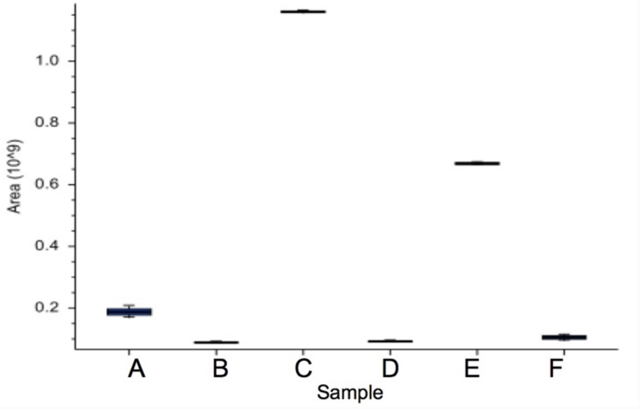

Gensentin

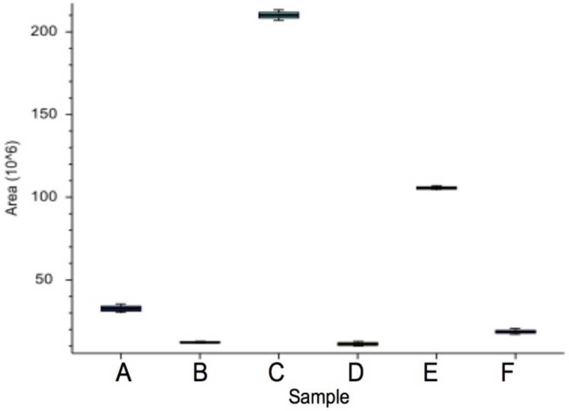

hispidulin

Compounds dominant in B and D

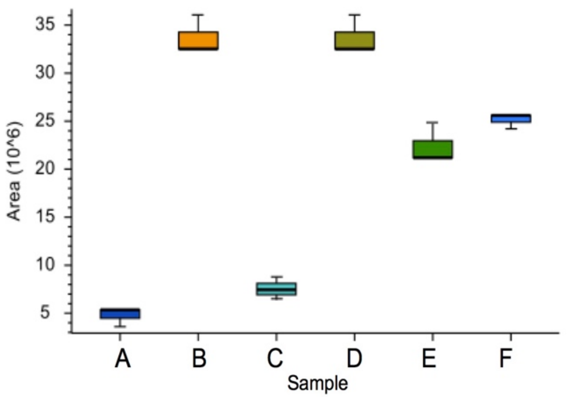

tricoumaroyl spermidine

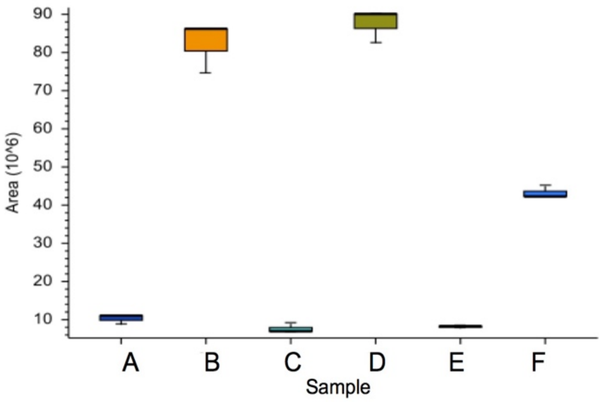

Cinnamic acid

Figure S2. Main metabolic pathway identified in isoflavonoids.

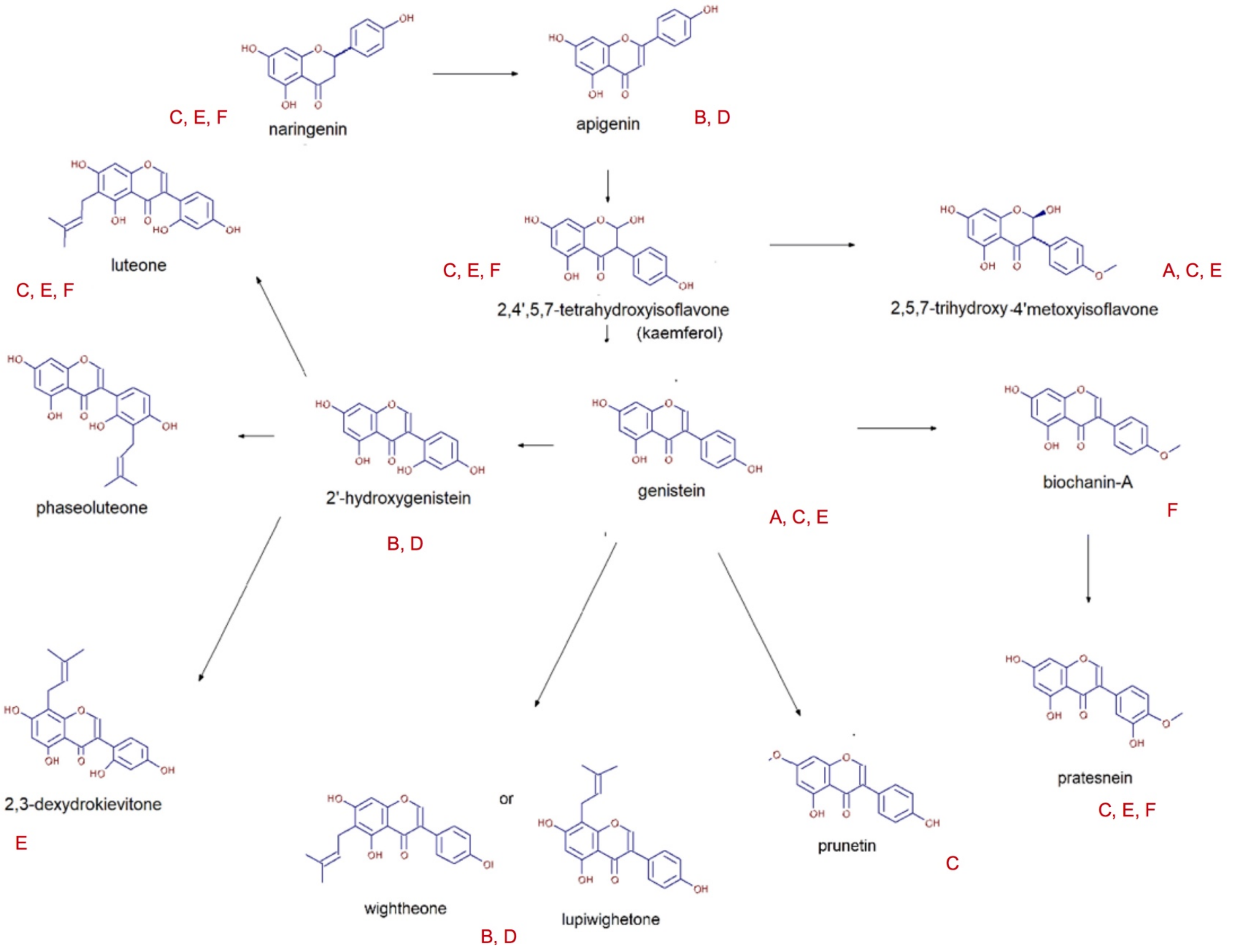

Supplement: Supplementary file 1 — Supplementary Information. [file 41598_2021_98876_MOESM1_ESM.pdf]
